# RCAN1 links impaired neurotrophin trafficking to aberrant development of the sympathetic nervous system in Down syndrome

**DOI:** 10.1038/ncomms10119

**Published:** 2015-12-14

**Authors:** Ami Patel, Naoya Yamashita, Maria Ascaño, Daniel Bodmer, Erica Boehm, Chantal Bodkin-Clarke, Yun Kyoung Ryu, Rejji Kuruvilla

**Affiliations:** 1 Department of Biology, Johns Hopkins University, 3400N. Charles Street, 224 Mudd Hall, Baltimore, Maryland 21218, USA

## Abstract

Down syndrome is the most common chromosomal disorder affecting the nervous system in humans. To date, investigations of neural anomalies in Down syndrome have focused on the central nervous system, although dysfunction of the peripheral nervous system is a common manifestation. The molecular and cellular bases underlying peripheral abnormalities have remained undefined. Here, we report the developmental loss of sympathetic innervation in human Down syndrome organs and in a mouse model. We show that excess regulator of calcineurin 1 (RCAN1), an endogenous inhibitor of the calcineurin phosphatase that is triplicated in Down syndrome, impairs neurotrophic support of sympathetic neurons by inhibiting endocytosis of the nerve growth factor (NGF) receptor, TrkA. Genetically correcting *RCAN1* levels in Down syndrome mice markedly improves NGF-dependent receptor trafficking, neuronal survival and innervation. These results uncover a critical link between calcineurin signalling, impaired neurotrophin trafficking and neurodevelopmental deficits in the peripheral nervous system in Down syndrome.

## Abstract (web summary)

Down syndrome is associated with both central and peripheral nervous system dysfunction. Here, the authors report reduced sympathetic neural innervation in human Down syndrome tissues and a mouse model, and propose a role for RCAN1 trisomy in disrupted NGF receptor trafficking and neurotrophic support.

## Graphical abstract

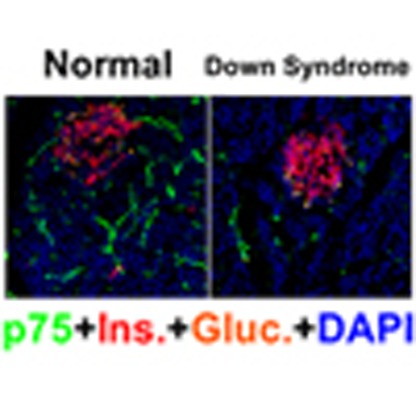

Down syndrome, a disorder that affects multiple tissues and results in delayed development and intellectual disability, is thought to originate from increased dosage of gene products encoded by human chromosome 21 (refs 1, 2, 3). To date, investigations of anomalies in the nervous system in Down syndrome have predominantly focused on the central nervous system (CNS) where cognitive impairment has been proposed to stem, in part, from abnormal brain development and an imbalance between excitatory and inhibitory neurotransmission^4^. However, individuals with Down syndrome also exhibit marked dysfunction of the peripheral nervous system^5^,^6^,^7^,^8^, the molecular and cellular bases of which remain undefined. The sympathetic nervous system is a branch of the autonomic nervous system that is essential for organ homeostasis. Postganglionic sympathetic neurons innervate diverse peripheral organs and tissues to govern fundamental physiological processes including regulation of blood glucose levels, cardiac output and body temperature. An emergent concept is that a dysfunctional sympathetic nervous system might be an instigating factor in the pathogenesis of congestive heart failure and diabetes^9^,^10^, diseases that are more prevalent in individuals with Down syndrome than in the general population^11^. In addition, individuals with Down syndrome have blunted cardiovascular responses to autonomic tasks including stress and exercise tests, and reduced secretion of the sympathetic neurotransmitter, norepinephrine^12^,^13^. Notably, impaired autonomic regulation of heart rate and blood pressure in the absence of congenital heart defects has been observed in Down syndrome^6^,^7^. Autonomic dysfunction in Down syndrome has been associated with reduced physical work capacity and chronic incompetence, manifested as attenuated heart rate responses to exercise^8^. In particular, chronotropic incompetence has been postulated to be predictive of coronary heart disease and early mortality^14^,^15^. Although impaired autonomic functions are manifested in infants and young children with Down syndrome^13^, it remains unknown whether functional deficits originate, in part, from aberrant development of the sympathetic nervous system.

Based on animal studies, the best characterized molecular player in sympathetic nervous system development is nerve growth factor (NGF), a neurotrophin that is secreted by peripheral tissues^16^. In newborn mice, genetic ablation of NGF or its cognate receptor, TrkA, results in diminished innervation of peripheral targets and loss of post-mitotic sympathetic neurons^17^,^18^,^19^, whereas transgenic overexpression of NGF in target tissues enhances sympathetic growth into final target fields^20^,^21^. As NGF is released by neuronal targets, a salient feature of NGF signalling in polarized neurons is the regulation of endocytic trafficking of its TrkA receptors and intracellular signalling from internalized receptors^22^. NGF promotes endocytosis of TrkA receptors in distal axons into NGF:TrkA-containing signalling endosomes that are retrogradely transported back to cell bodies to exert transcriptional control of neuronal survival and long-term growth^23^. Although the functional relevance of neurotrophin trafficking has been most appreciated during normal development, a corollary view is that dysregulation of endocytic trafficking could be the basis for decreased neurotrophic support in developmental disorders and late-onset neurodegenerative diseases^22^,^24^,^25^. However, little is known about molecular mechanisms that impair neurotrophin trafficking in a disease state.

Here, we report a pronounced loss of sympathetic innervation in a mouse model of Down syndrome and human Down syndrome tissues. We identify an underlying mechanism that links perturbed endocytic trafficking of NGF receptors to developmental defects in neuronal survival and axon growth. Our findings implicate deficient calcineurin phosphatase signalling through overexpression of *regulator of calcineurin 1* (*RCAN1*), an endogenous calcineurin inhibitor that is triplicated in Down syndrome, in contributing to aberrant development by interfering with receptor endocytosis and retrograde trophic signalling. Furthermore, using transgenic mice trisomic for *RCAN1* alone as well as genetically correcting *RCAN1* levels in Down syndrome mice, we establish a causal link between increased *RCAN1* dosage, impaired neurotrophin receptor trafficking and developmental abnormalities in the sympathetic nervous system in Down syndrome.

## Results

### Sympathetic innervation is reduced in down syndrome tissues

To investigate the sympathetic nervous system in Down syndrome, we employed a mouse model of Down syndrome, *Dp(16)1Yey/+* mice, that harbour a 22.9-Mb duplication spanning the entire region of mouse chromosome 16 syntenic with human chromosome 21 (ref. 26). *Dp(16)1Yey/+* mice are trisomic solely for the human 21q11-q22.3 syntenic region compared with the widely used Ts65Dn model, and exhibit cognitive, cardiovascular and gastrointestinal phenotypes recapitulating that observed in humans with Down syndrome^26^,^27^,^28^. We performed whole-mount immunostaining for tyrosine hydroxylase (TH), a marker for noradrenergic neurons, to examine the formation of the entire sympathetic chain ganglia and their axonal projections. We examined mice at embryonic day 16.5 (E16.5), a stage when neurogenesis, migration and noradrenergic specification are completed in the murine sympathetic nervous system^18^. We observed that sympathetic chain ganglia had coalesced into discrete condensations, with axonal projections coursing along the intercostal arteries in both *Dp(16)1Yey/+* and litter-mate embryos (Supplementary Fig. 1a,b). In addition, TH immunohistochemistry and quantification of cell numbers using Nissl staining in tissue sections revealed no significant differences in size, shape and neuronal numbers in the superior cervical ganglia (SCG), the rostral-most ganglia in the sympathetic chain, between E16.5 *Dp(16)1Yey/+* and wild-type embryos (Supplementary Fig. 1c–e). Therefore, early developmental processes including neuronal production, migration and specification in the sympathetic nervous system are unaffected in Down syndrome mice.

**Figure 1:**
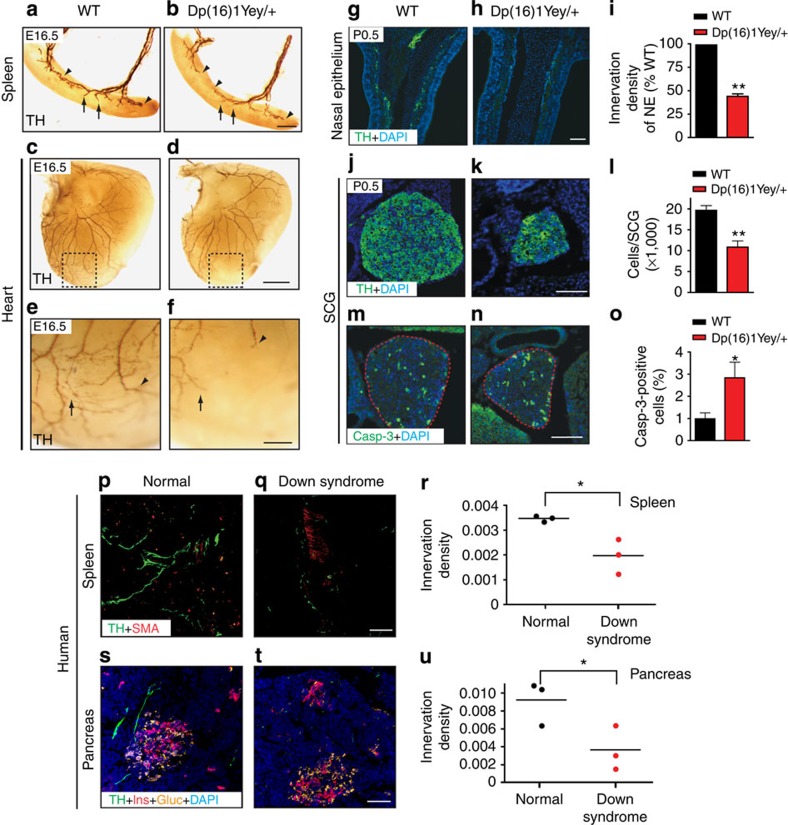
Decreased sympathetic innervation in *Dp(16)1Yey/+* mice and human Down syndrome tissues. (**a**–**f**) Sympathetic innervation of target organs is decreased in *Dp(16)1Yey/+* embryos. Whole-mount tyrosine hydroxylase (TH) immunostaining of the spleen (**a**,**b**) and heart (**c**,**d**) reveals that axons are shorter, thinner and less branched in E16.5 *Dp(16)1Yey/+* mice compared with litter-mate controls. Higher magnification images of the heart are shown in **e**,**f**. Terminal extension and branching within the target fields are indicated by arrowheads and arrows, respectively. Representative images are shown from at least three animals per genotype that were analysed. Scale bar, 200 μm for **a**,**b**, 500 μm for **c**,**d** and 200 μm for **e**,**f**. (**g**–**i**) Reduced sympathetic innervation of nasal epithelium in *Dp(16)1Yey/+* mice compared with wild-type litter-mates at birth (P0.5), determined by TH immunohistochemistry of tissue sections. DAPI staining is included to reveal the cellular material in the entire tissue section. Values are the mean±s.e.m., *n*=3 for each genotype, **P<0.01, Scale bar, 50 μm. (**j**–**l**) Decreased sympathetic ganglia size and cell numbers in P0.5 *Dp(16)1Yey/+* mice. SCGs were visualized by TH immunohistochemistry and cell counts were performed on Nissl-stained tissue sections. Values are the mean±s.e.m., *n*=3 mice for wild-type and *n*=4 for *Dp(16)1Yey/+* mice. **P<0.01. Scale bar, 100 μm. (**m**–**o**) Increased apoptosis in P0.5 *Dp(16)1Yey/+* SCGs, assessed by cleaved caspase-3 immunostaining. SCGs are outlined in dashed lines. Values are the mean±s.e.m., *n*=4 mice for each genotype. *P<0.05. Scale bar: 100 μm. Statistical analyses by unpaired two-tailed Student's *t*-test for (**i**,**l**,**o**). (**p**–**u**) Diminished sympathetic innervation of Down syndrome peripheral tissues. TH immunostaining (in green) shows reduced sympathetic innervation of the spleen (**p**,**q**) and pancreatic tissues (**s**,**t**) from children with Down syndrome relative to normal individuals. Spleen tissue sections were immunostained with smooth muscle actin (SMA, red) to reveal blood vessels, and pancreatic tissues were immunostained with insulin (in red, Ins) and glucagon (in orange, Gluc). Scale bar, 50 μm. (**r**,**u**) Quantification of TH-positive sympathetic fibres by measuring the integrated fluorescent density per unit area using ImageJ. Results were expressed as fluorescence units per 100 μm^2^. Human tissues were obtained from three Down syndrome children and three age-and gender-matched controls. *P<0.05, one-tailed *t*-test.

Examination of innervation of distal target fields, however, revealed a marked impairment in E16.5 *Dp(16)1Yey/+* embryos. Sympathetic fibres reached and innervated end-organs such as the spleen, heart and nasal epithelium in *Dp(16)1Yey/+* embryos, but the axons were shorter, sparser and less branched within the target fields (Fig. 1a–f and Supplementary Fig. 1f–h). Further examination of innervation at post-natal day 0.5 (P0.5) revealed fewer sympathetic fibres in the nasal epithelium (Fig. 1g–i) and salivary glands (Supplementary Fig. 1i–k) in *Dp(16)1Yey/+* mice, compared with control litter-mates. Quantification of SCG neuronal numbers revealed a substantial 45% decrease in *Dp(16)1Yey/+* mice at P0.5 (Fig. 1j–l), and a concomitant increase in apoptotic profiles (Fig. 1m–o).

Given these developmental abnormalities in the mouse model of Down syndrome, we then assessed sympathetic innervation of peripheral organs from Down syndrome infants (see Supplementary Table 1 for specimen details). Human spleen and pancreatic tissues were subjected to TH immunostaining. Compared with control tissues, we observed a pronounced decrease of TH-positive fibres in the Down syndrome spleen (Fig. 1p–r) and pancreatic (Fig. 1s–u) tissues. Quantification revealed a significant reduction in TH immunoreactivity in the Down syndrome tissues compared with controls (*P*=0.01 for spleen, *P*=0.026 for pancreas, *t*-test, *n*=3 each for Down syndrome and normal donor tissues). Co-labelling with antibodies against smooth muscle actin to mark the spleen vasculature (Fig. 1p,q and Supplementary Fig. 2a,b), and insulin and glucagon to identify pancreatic islets (Fig. 1s,t and Supplementary Fig. 2f,g), showed no overall disruptions in tissue morphology in the Down syndrome samples. Furthermore, haematoxylin and eosin staining revealed intact tissue structure and similar histology between the Down syndrome and control tissues (Supplementary Fig. 2d,e,i,j). To address if the innervation defect in Down syndrome tissues was specific to sympathetic axons, we performed immunostaining for neurofilament, a pan-neuronal marker. We observed a trend towards decreased neurofilament immunoreactivity in the Down syndrome spleen (Supplementary Fig. 2a–c) and pancreas (Supplementary Fig. 2f–h), although these deficits were not as severe as the decreases in TH immunoreactivity, and were not statistically different from control tissues. This is likely due to sympathetic nerves constituting only a fraction of total peripheral innervation. Double labelling revealed that ∼30% of the neurofilament-immunoreactive fibres innervating the human spleen and pancreatic tissues are TH positive (Supplementary Fig. 2q,x). Notably, we observed a significant reduction in the amount of TH/neurofilament-double-positive axons in Down syndrome tissues (Supplementary Fig. 2k–x). Together, these findings provide evidence of aberrant sympathetic nervous system development in human Down syndrome tissues, analogous to that in *Dp(16)1Yey/+* mice.

### Trk endocytosis is attenuated in *Dp(16)1Yey*/+ neurons

**Figure 2:**
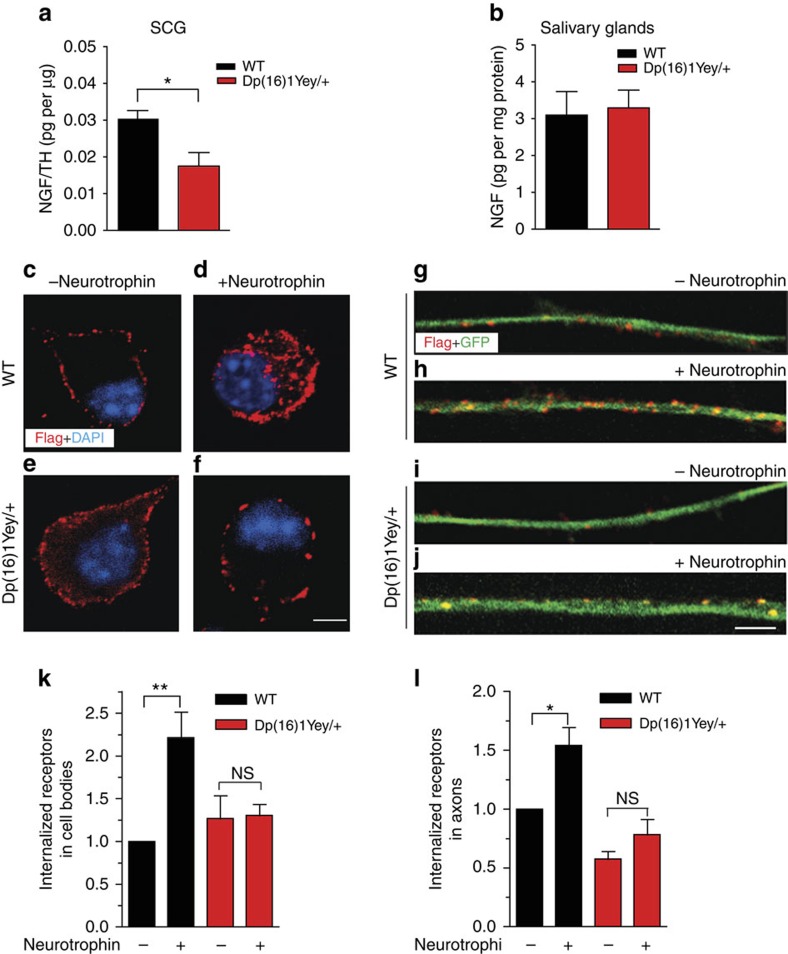
Impaired ligand-dependent endocytosis of Trk receptors in *Dp(16)1Yey/+* neurons. (**a**,**b**) *Dp(16)1Yey/+* mice show a significant decrease in NGF protein levels in sympathetic cell bodies located in superior cervical ganglia (SCG; **a**), but not in the salivary glands (**b**), a target tissue innervated by sympathetic axons. NGF levels in SCGs were normalized to TH in SCGs and represented as picograms of NGF per μg of TH. NGF levels in salivary glands were normalized to total protein. Results are the mean±s.e.m. from *n*=3 *Dp(16)1Yey/+* mice and four control litter-mates for SCGs, and *n*=5 mice per genotype for salivary glands. *P<0.05, unpaired two-tailed Student's *t*-test. (**c**–**j**) Ligand-dependent Trk receptor internalization is impaired in *Dp(16)1Yey/+* sympathetic neurons. Sympathetic neurons from P0.5 *Dp(16)1Yey/+* and wild-type (WT) sympathetic neurons were established in microfluidic chambers and infected with an adenovirus expressing FLAG-TrkB:A chimeric receptors. Neurons were labelled with FLAG antibody under non-permeabilizing conditions at 4 °C for 30 min, followed by BDNF treatment for 30 min. FLAG immunoreactivity (red) was assessed in cell bodies (**c**–**f**) and axons (**g**–**j**). Scale bars, 5 and 10 μm for axons and cell bodies, respectively. (**k**,**l**) Quantification of internalized Trk in cell bodies and axons after treatments described in **c**–**j**. Internal accumulation of chimeric receptors under the various conditions was determined by assessing the proportion of co-localization of FLAG immunofluorescence with that of GFP, which is co-expressed in infected neurons and is cytoplasmic. At least 40–50 neurons were analysed per condition. Quantification is represented as fold-change relative to WT neurons with no neurotrophin. Results are the mean±s.e.m. from three independent experiments. *P<0.05, **P<0.01, NS, not significant, two-way analysis of variance (ANOVA) followed by Bonferroni *post-hoc* test.

The deficits in sympathetic innervation in *Dp(16)1Yey/+* embryos at E16.5 were reminiscent of phenotypes observed in mice lacking the target-derived neurotrophin, NGF^19^,^29^, which is known to control axonal extension and arborization when sympathetic axons have reached their final destinations^16^,^29^. The neuronal loss observed at P0.5, when sympathetic axons are actively engaged in a developmental survival competition for limiting amounts of NGF^16^, and the magnitude of the loss, similar to that in new-born *TrkA*^*−/−*^ mice^18^, further implicate a failure in NGF signalling in *Dp(16)1Yey*/+ mice. Using an ELISA-based immunoassay, we found a significant 43.5% decrease in NGF protein levels in SCG lysates from *Dp(16)1Yey/+* mice (Fig. 2a). However, NGF levels in the salivary glands were similar to that in wild-type mice (Fig. 2b). The salivary glands are the primary source of NGF for the SCG^30^; NGF produced in the salivary glands binds to TrkA receptors on sympathetic axons, and following endocytosis, is retrogradely transported to cell bodies located within the SCG. Similar to salivary glands, normal NGF levels were also found in another sympathetic target organ, the heart, in *Dp(16)1Yey/+* mice (Supplementary Fig. 3). These results suggest that diminished innervation and neuronal loss in *Dp(16)1Yey/+* mice arise from deficits in NGF uptake and/or retrograde transport in sympathetic neurons rather than decreased NGF production in target tissues.

NGF-mediated survival and axon growth of sympathetic neurons is critically dependent on endocytosis of TrkA receptors, the primary event in retrograde NGF signalling^31^,^32^. Given the decrease in NGF protein accumulation in sympathetic neuron cell bodies in *Dp(16)1Yey/+* mice, we examined endocytic trafficking of Trk receptors using a chimeric Trk receptor-based, live-cell antibody feeding assay in cultured neurons^33^. Cultured sympathetic neurons isolated from P0.5 *Dp(16)1Yey/+* and wild-type mice were infected with an adenoviral vector expressing FLAG-tagged chimeric receptors that have the extracellular domain of TrkB and the transmembrane and intracellular domains of TrkA (FLAG-TrkB:A). Sympathetic neurons do not normally express TrkB receptors, and the chimeric Trk receptors respond to the TrkB ligand, brain-derived neurotrophic factor (BDNF), but retain the signalling properties of TrkA^33^. Using live-cell immunocytochemistry with an antibody directed against the extracellular FLAG epitope, we observed prominent receptor internalization in response to neurotrophin stimulation in both the cell bodies (Fig. 2c,d) and axons (Fig. 2g,h) in wild-type neurons. In contrast, ligand-dependent internalization was markedly attenuated in *Dp(16)1Yey/+* neurons (Fig. 2e,f,i,j). Although neurotrophin treatment enhanced Trk receptor internalization by 2.2±0.2-fold in cell bodies, and 1.6±0.15-fold in axons in wild-type neurons, there were no significant differences between the un-stimulated and ligand-treated conditions in *Dp(16)1Yey/+* neurons (Fig. 2k,l). Together, these results indicate that ligand-dependent endocytosis of Trk receptors is attenuated in *Dp(16)1Yey/+* sympathetic neurons.

### Excess RCAN1 inhibits TrkA endocytosis

*Dp(16)1Yey/+* mice are trisomic for 113 genes orthologous to human chromosome 21 (refs 26, 28). Increased dosage of one or more of these genes could serve as the molecular locus for impaired Trk receptor trafficking and the aberrant development of the sympathetic nervous system. A clue to the identity of the responsible gene came from our previous observations that the calcium-calmodulin-activated phosphatase, calcineurin, is necessary for NGF-dependent endocytosis of TrkA receptors, and for sympathetic innervation of target tissues^34^. Among the 113 trisomic genes in *Dp(16)1Yey/+* mice is *RCAN1*, originally named *Down's syndrome candidate region 1* (*DSCR1*) because of the gene locus within human chromosome 21 (refs 35, 36). RCAN1 belongs to a family of endogenous calcineurin inhibitors that are highly conserved from yeast to humans^37^,^38^,^39^,^40^,^41^,^42^,^43^. RCAN1 expression is enriched in tissues that are particularly vulnerable in Down syndrome, including the nervous system, heart and skeletal muscle, and its levels are significantly elevated in individuals with Down syndrome and mouse models^35^,^36^,^38^. We observed a significant (58%) decrease in calcineurin phosphatase activity in SCG lysates from P0.5 *Dp(16)1Yey/+* mice (see Fig. 7b for details). Thus, we hypothesized that dysfunctional calcineurin signalling through increased *RCAN1* dosage is a potential link between deficits in TrkA trafficking and decreased neurotrophic support in Down syndrome.

**Figure 3:**
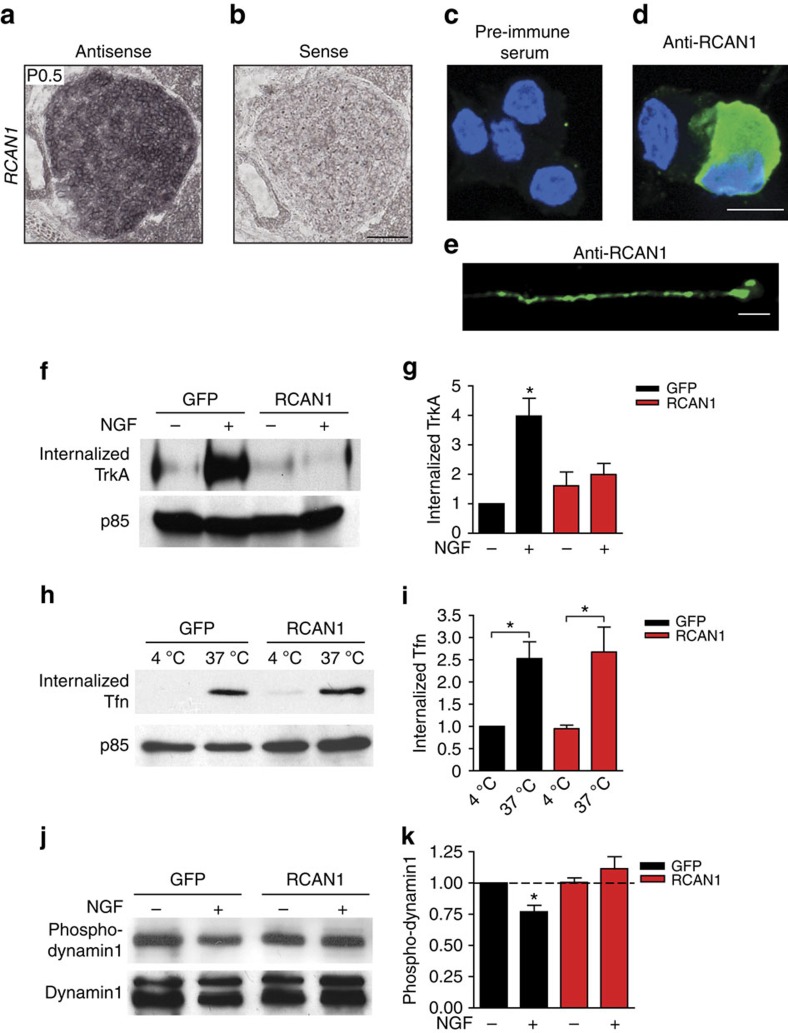
Increased expression of RCAN1, an endogenous calcineurin inhibitor, downregulates TrkA endocytosis by altering dynamin1 phosphorylation. (**a**) *In situ* hybridization shows endogenous expression of *RCAN1* mRNA in the developing mouse superior cervical ganglia at P0.5. Sense control is shown in **b**. Scale bar: 100 μm. (**c**–**e**) RCAN1 protein is localized to both cell bodies (**d**) and axons (**e**) of cultured sympathetic rat neurons as detected using a RCAN1 antibody. Staining with pre-immune serum control is shown in **c**. Scale bar, 10 μm for **c**,**d** and 5 μm for **e**. (**f**) A cell surface biotinylation assay shows that adenoviral overexpression of RCAN1.4 attenuates NGF-dependent TrkA internalization in cultured rat sympathetic neurons. Membrane proteins were subjected to cell-surface biotinylation. Internalized TrkA receptors were detected by surface stripping of biotin, neutravidin precipitation and TrkA immunoblotting. Supernatants were probed for p85 for normalization of protein amounts. (**g**) Densitometric quantification of internalized TrkA. Results are means±s.e.m. from four independent experiments. *P<0.05 significantly different from all other conditions. (**h**) Uptake of biotin-labelled transferrin (biotin-Tfn) is unaffected by RCAN1 overexpression in rat sympathetic neuron cultures. After internalization at 37 °C and acid washes to remove surface-bound transferrin, internalized biotin-Tfn was detected in neuronal lysates by neutravidin precipitation and immunoblotting using a transferrin antibody. Supernatants were probed for p85 for normalization of protein amounts. (**i**) Densitometric quantification of internalized biotin-Tfn. Results are means±s.e.m. from five independent experiments. *P<0.05 significantly different from corresponding controls at 4 °C. (**j**) NGF stimulation results in dephosphorylation of dynamin1, that is abrogated by excess RCAN1. Neuronal lysates were immunoblotted using a phospho-Ser778 dynamin antibody. Immunoblots were stripped and reprobed for total dynamin1 for normalization. (**k**) Densitometric quantification of phospho-dynamin1 levels normalized to total dynamin1 levels. All values are expressed relative to the no neurotrophin treatment in GFP-expressing neurons. Results are means±s.e.m. from seven independent experiments. *P<0.05 significantly different from all other conditions. Statistical analyses by two-way ANOVA and Bonferroni *post-hoc* test for **g**,**i**,**k**. Full-length blot scans are shown in Supplementary Fig. 8.

As a first step towards defining the role of RCAN1 in NGF-dependent functions, we assessed the expression of endogenous *RCAN1* in sympathetic neurons. Differential promoter usage results in two different *RCAN1* transcripts, *RCAN1.1* and *RCAN1.4*, where exons 1 or 4 are alternatively used as the first exon followed by the same shared last three exons (Supplementary Fig. 4a)^36^,^44^. Reverse transcription–PCR (RT–PCR) analyses revealed that both *RCAN1.1* and *RCAN1.4* isoforms are present in developing SCGs in newborn mice (Supplementary Fig. 4b). *In situ* hybridization using a probe directed against a common region in the two *RCAN1* isoforms showed *RCAN1* expression in the SCG (Fig. 3a,b) and in another NGF-responsive neuronal population, dorsal root sensory ganglia (DRG), at postnatal day 0.5 (P0.5; Supplementary Fig. 4c,d). Immunostaining in dissociated sympathetic neurons revealed that RCAN1 protein is localized throughout the neuron (Fig. 3c–e).

We next reasoned that excess RCAN1 would interfere with TrkA endocytosis. Thus, a cell surface biotinylation assay was performed to measure NGF-dependent internalization of endogenous TrkA receptors in sympathetic neurons infected with an adenoviral vector expressing HA-tagged human RCAN1.4. Adenovirus-mediated expression resulted in an elevation of RCAN1 protein levels by 2.8-fold (Supplementary Fig. 4e,f). Treatment of sympathetic neurons with NGF for 30 min elicited robust internalization of TrkA receptors in control neurons expressing green fluorescent protein (GFP). In contrast, TrkA internalization was markedly reduced in RCAN1-overexpressing neurons (Fig. 3f,g). Similarly, NGF-dependent endocytosis of TrkA receptors was also blunted in DRG neurons infected with RCAN1 adenovirus (Supplementary Fig. 4g,h). To determine if the endocytosis defect with excess RCAN1 was specific to TrkA receptors, we examined the endocytosis of the Transferrin receptor (TfR), a prototypical constitutively internalized receptor^45^. Sympathetic neurons were incubated with biotin-transferrin or Alexa-555-labelled-transferrin at 4 °C and intracellular transferrin accumulation at 37 °C was either monitored biochemically by streptavidin precipitation followed by transferrin immunoblotting, or by following uptake of the fluorescent label, respectively. There were no significant differences in TfR internalization between RCAN1- and GFP-expressing sympathetic neurons (Fig. 3h,i and Supplementary Fig. 4i–m). Similarly, the uptake of Alexa-555-labelled epidermal growth factor (EGF) was also unaffected in RCAN1-overexpressing neurons (Supplementary Fig. 4n–r), suggesting normal internalization of EGF receptor, a receptor tyrosine kinase that undergoes ligand-induced internalization^45^.

To identify the molecular mechanisms by which RCAN1 overexpression impairs TrkA endocytosis, we focused on the endocytic GTPase, dynamin1. Previously, we found that calcineurin dephosphorylates neuron-specific splicing isoforms of dynamin1 to drive TrkA internalization in sympathetic neurons^34^. Therefore, we assessed the phosphorylation status of dynamin1 in response to NGF in neurons overexpressing RCAN1 or GFP. Sympathetic neurons were exposed to NGF for 30 min, and levels of phosphorylated dynamin1 assessed using a phospho-specific antibody that specifically recognizes dynamin1 phosphorylated on Ser-778, one of two sites known to be dephosphorylated by calcineurin^46^. NGF treatment induced a significant decrease in dynamin1 phosphorylation (by 22%) in control GFP-expressing neurons (Fig. 3j,k). This decrease in dynamin1 phosphorylation was abolished by RCAN1 overexpression (Fig. 3j,k). These findings indicate that excess RCAN1 disrupts internalization of TrkA receptors by inhibiting calcineurin-mediated dephosphorylation of dynamin1.

### RCAN1 overexpression attenuates retrograde NGF signalling

**Figure 4:**
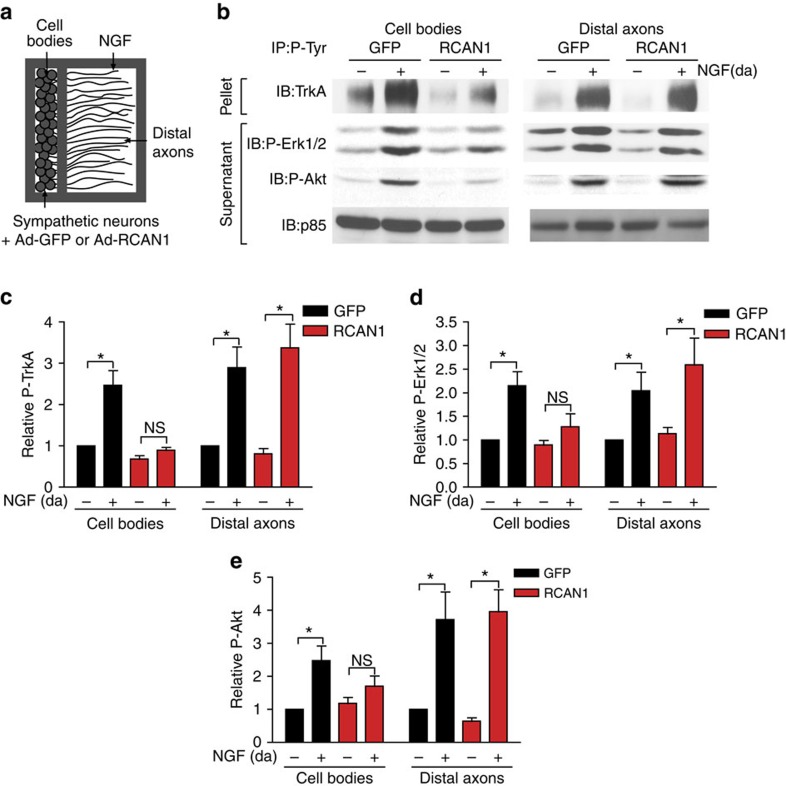
RCAN1 overexpression attenuates retrograde NGF signalling. (**a**) Schematic of the compartmentalized culture system used for biochemical analyses of NGF-dependent signalling locally in distal axons and retrogradely in cell bodies. (**b**) NGF stimulation of distal axons promotes phosphorylation of P-TrkA, P-Erk1/2 and P-Akt locally in axons and retrogradely in cell bodies of control GFP-expressing sympathetic neurons. RCAN1 overexpression disrupts the propagation of a retrograde NGF signal to cell bodies but does not affect local activation of these effectors in distal axons. Distal axons (da) of sympathetic neurons expressing GFP or RCAN1 were stimulated with NGF (100 ng per ml) for 8 h. Cell body/proximal axon and distal axon lysates were prepared and subjected to immunoprecipitation with a P-Tyrosine (PY20) antibody followed by immunoblotting for TrkA to detect P-TrkA. Supernatants were immunoblotted for P-Erk1/2, P-Akt and p85. (**c**–**e**) Densitometric quantifications of levels of P-TrkA (**c**), P-Erk1/2 (**d**) and P-Akt (**e**). P-TrkA, P-Erk1/2 and P-Akt signals were all normalized to p85 levels. Results are means±s.e.m. from five independent experiments, and expressed relative to no neurotrophin conditions. NS, not significant, *P<0.05 by two-way ANOVA and Bonferroni test. Full-length blot scans are shown in Supplementary Fig. 8.

**Figure 5:**
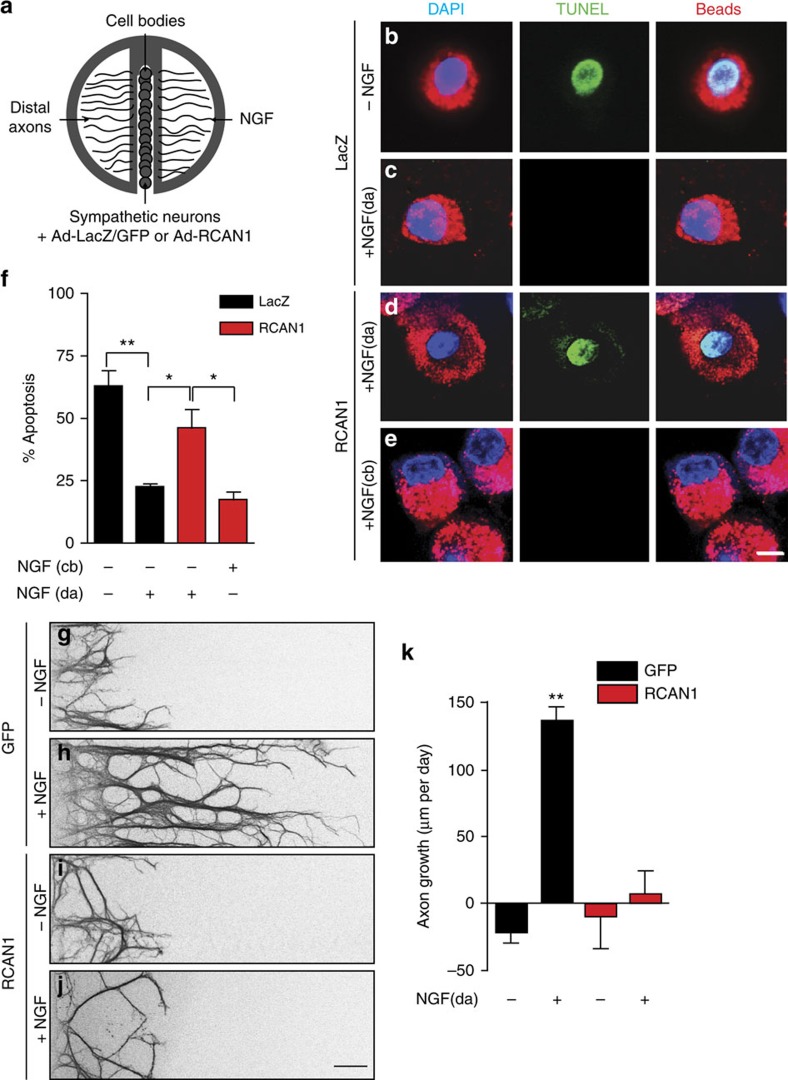
Excess RCAN1 perturbs NGF-dependent trophic support of sympathetic neurons. (**a**) Sympathetic neurons were grown in compartmentalized cultures, infected with RCAN1 or control LacZ or GFP adenoviruses, and NGF-dependent neuronal survival and axon growth was measured. (**b**) TUNEL labelling (green) indicates neuronal apoptosis in the presence of a neutralizing NGF antibody (anti-NGF, added to both cell body and axonal compartments). (**c**) Neuronal survival with NGF (100 ng per ml) present only on distal axons (da) in control LacZ-infected neurons. (**d**,**e**) RCAN1 overexpression compromises neuronal survival when NGF is present on distal axons (da) but not when NGF is added directly to cell bodies (cb). Neuronal apoptosis was assessed in neurons that had extended axons into the side chambers, visualized by retrograde accumulation of fluorescent microspheres (red). Neuronal nuclei are labelled with DAPI (blue). Scale bar, 10 μm. (**f**) Neuronal apoptosis was calculated by determining the percentage of projecting neurons that were TUNEL-positive. *P<0.05, **P<0.01, results are means±s.e.m. from four independent experiments. (**g**–**j**) NGF-dependent axon growth is abolished by RCAN1 overexpression. Compartmentalized cultures of sympathetic neurons expressing GFP or RCAN1 were either deprived of NGF by including anti-NGF (1:1,000) in media bathing cell body and axon compartments (**g**,**i**), or maintained with NGF (100 ng per ml) added solely to the axonal compartments (**h**,**j**). The caspase inhibitor, BAF (50 μM), was included in all experiments to prevent cell death. Panels in **g**–**j** are representative images of axons immunostained with anti-β-III tubulin 24 h after addition of anti-NGF or NGF to distal axons. Scale bar, 50 μm. (**k**) Quantification of axon growth in compartmentalized cultures. Rate of axon extension (μm per day) was assessed in 24 h intervals for a total of 72 h. Results are means±s.e.m. from five independent experiments. **P<0.01 different from all other conditions. One-way ANOVA followed by Tukey's *post-hoc* test for **f**, and two-way ANOVA and Bonferroni *post-hoc* test for **k**.

The finding that RCAN1 overexpression abrogated TrkA endocytosis led us to predict that excess RCAN1 would impact NGF-mediated retrograde communication between axon terminals and distal neuronal cell bodies, a process that relies on TrkA endocytosis within nerve terminals. To assess retrograde NGF signalling in neurons, we used a compartmentalized culture system that allows for the separation of cell bodies and proximal axons from the distal axons by a teflon-grease diffusion barrier, and the application of neurotrophins exclusively to distal axons, thus recapitulating the *in vivo* situation (Fig. 4a, also see Fig. 5a). In compartmentalized cultures, NGF treatment of distal axons (100 ng per ml, 8 h) resulted in the robust phosphorylation of TrkA receptors and of canonical signalling effectors, Erk1/2 and Akt, locally within distal axons in both RCAN1- and GFP-expressing neurons. However, although NGF promoted the retrograde accumulation of P-TrkA, P-Erk1/2 and P-Akt in cell bodies of control neurons, there was a pronounced reduction in the levels of these signalling molecules in the cell bodies of RCAN1-expressing neurons (Fig. 4b–e).

If RCAN1 effects are mediated via its modulation of calcineurin activity, then local inhibition of calcineurin activity in distal axons should perturb retrograde NGF signalling. Indeed, we found that addition of the pharmacological calcineurin inhibitors, Cyclosporin A (CsA; 2 μg per ml) plus FK506 (0.2 μg per ml), exclusively to distal axons attenuated NGF-dependent retrograde signalling in compartmentalized cultures (Supplementary Fig. 5a–d). Analogous to the findings with RCAN1 overexpression, axonal application of calcineurin inhibitors had no effect on NGF-dependent phosphorylation of TrkA, Erk1/2 and Akt locally within distal axons. Together, these results indicate that RCAN1 overexpression or inhibition of calcineurin activity in axons interferes with the long-distance retrograde propagation of the NGF signal.

### Excess RCAN1 disrupts NGF-dependent trophic functions

Retrograde propagation of the NGF signal is a prerequisite step in the ability of target-derived NGF to support neuronal survival^31^,^32^. Thus, we asked if RCAN1 overexpression would compromise the ability of axon-applied NGF to retrogradely support neuronal survival. We monitored neuronal survival in response to NGF (100 ng per ml) added exclusively to distal axons in compartmentalized cultures of sympathetic neurons infected with the RCAN1 or a control LacZ adenovirus (Fig. 5a). In LacZ-infected neurons, NGF was sufficient to support the survival of the majority of neurons with only ∼22.7±1.2% undergoing apoptosis, assessed by TUNEL staining (Fig. 5b,c,f). In contrast, RCAN1-overexpressing neurons exhibited a significant increase in neuronal apoptosis (46.4±7.4% apoptotic neurons; Fig. 5d,f). Notably, NGF added directly to neuronal cell bodies in compartmentalized cultures is known to promote survival by an endocytosis-independent mechanism^47^, and in this condition, RCAN1 overexpression did not elicit increased apoptosis (17.6±3.2% apoptotic neurons; Fig. 5e,f). Thus, the overexpression of RCAN1 specifically compromises neuronal survival when NGF is present on distal axons, a scenario where endocytic trafficking of TrkA receptors is essential for retrograde NGF survival signalling.

We then sought to determine if RCAN1 overexpression would influence NGF-dependent axon growth, a process that is also dependent on calcineurin-mediated endocytosis of TrkA receptors^34^. In these analyses, the broad-spectrum caspase inhibitor, boc-aspartyl(O-methyl)-fluoromethylketone (BAF, 50 μM), was added to cell bodies of compartmentalized cultures so that axon growth could be assessed independent of complications of RCAN1-mediated apoptosis. NGF (100 ng per ml) added only to distal axons promoted robust growth in control neurons, with an average growth rate of 136 μm per day (Fig. 5g,h,k,). In contrast, the growth-promoting effect of NGF was abolished in RCAN1 overexpressing neurons (Fig. 5i–k).

### *RCAN1* triplication perturbs sympathetic development

**Figure 6:**
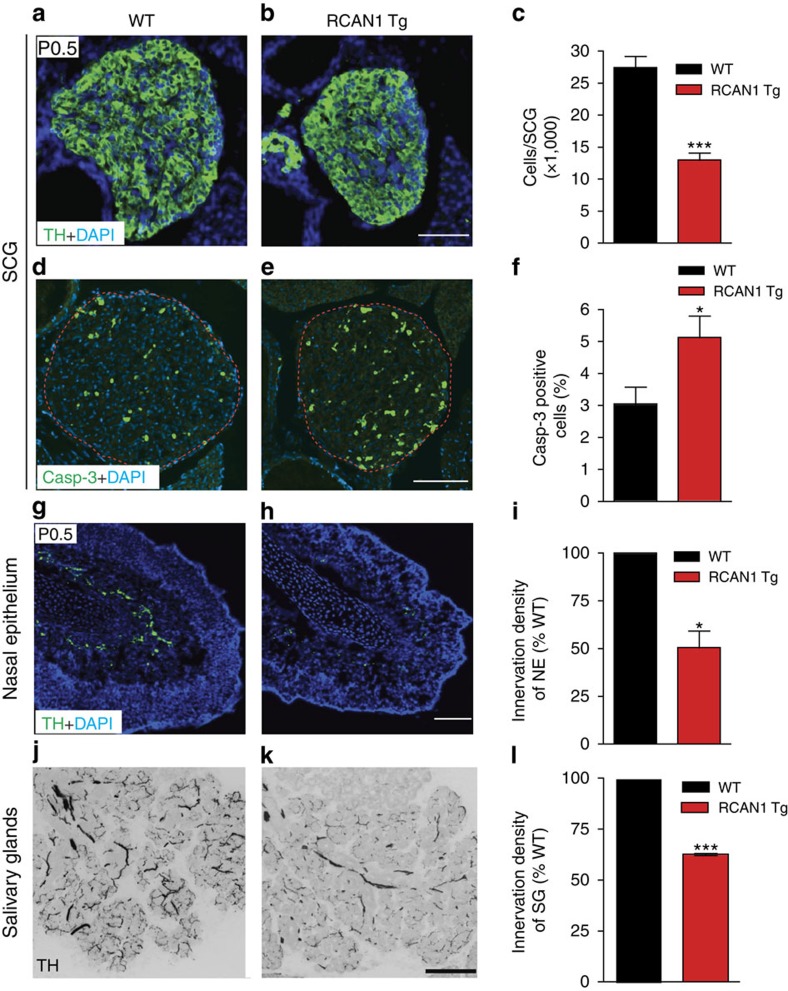
*RCAN1* transgenic mice exhibit loss of sympathetic neurons and reduced sympathetic innervation of target tissues. (**a**–**c**) Transgenic mice expressing three copies of *RCAN1* (*RCAN1* Tg) exhibit significant decreases in SCG size and cell numbers compared with litter-mate controls (WT) at P0.5. SCGs were visualized using TH immunohistochemistry and cell counts performed on Nissl-stained tissues. Values are the mean±s.e.m., *n*=5 mice each for wild-type and *RCAN1* Tg mice. ***P<0.001. (**d**,**e**) Cleaved caspase-3 immunofluorescence shows enhanced apoptosis in P0.5 SCGs from *RCAN1* Tg mice. (**f**) Quantification of percentage of SCG neurons that were immunoreactive for caspase-3. Values are the mean±s.e.m., *n*=5 mice for each genotype. *P<0.05. (**g**–**l**) TH immunostaining of sympathetic target tissues show substantial reductions in TH-positive sympathetic fibres within the nasal epithelium (**g**–**i**) and salivary glands (**j**–**l**) in *RCAN1* Tg mice compared with litter-mate controls, at P0.5. For quantification of innervation density, the ratio of TH immunoreactivity to total image area was calculated from multiple images. The results are represented as a percentage of the mean for wild-type mice for nasal epithelium (**i**) and salivary glands (**l**). Values are the mean±s.e.m., *n*=3 mice for each genotype. *P<0.05, ***P<0.001. Statistical analyses by unpaired two-tailed Student's *t*-test. Scale bars, 100 μm.

**Figure 7:**
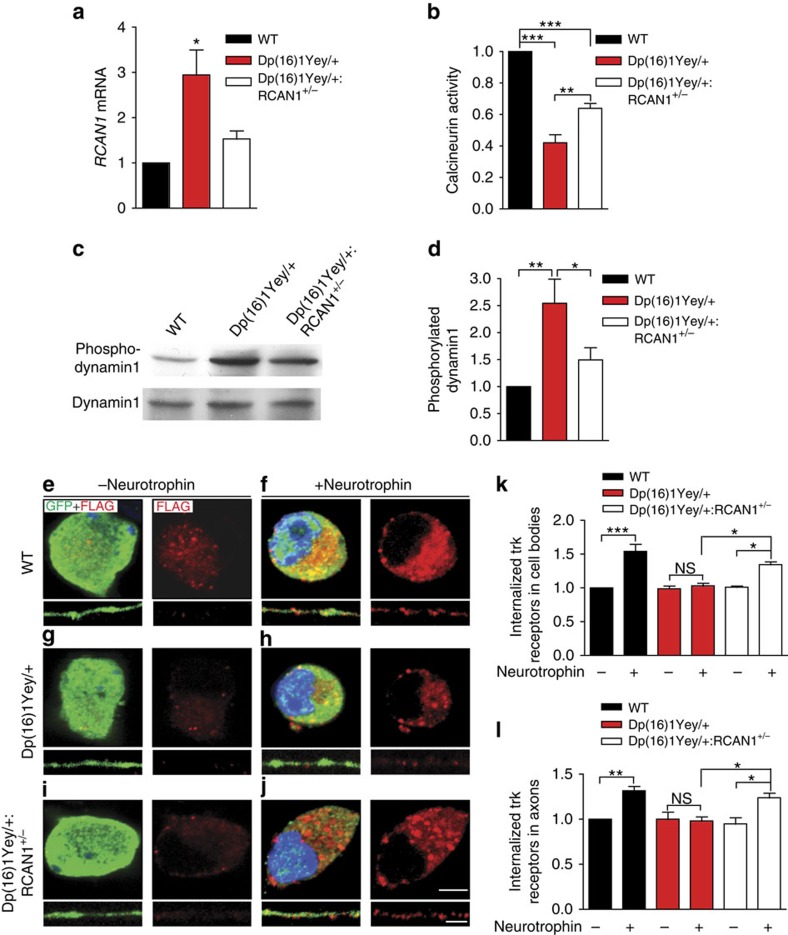
Reducing *RCAN1* gene dosage ameliorates defects in calcineurin activity, dynamin phosphorylation and Trk receptor trafficking in *Dp(16)1Yey/+* mice. (**a**) Quantitative RT–RCR shows a 2.9-fold increase in *RCAN1* mRNA in SCGs from *Dp(16)1Yey/+* mice compared with wild-type litter-mates. *RCAN1* levels are reduced in *Dp(16)1Yey/+:RCAN*^*+/−*^ mice that are diploid for *RCAN1* and trisomic for the rest of human chromosome 21 syntenic region on mouse chromosome 16. Results are mean±s.e.m., *n*=7 mice for each genotype. *P<0.05 different from all other conditions. (**b**) Calcineurin activity is significantly reduced in SCG lysates from P0.5 *Dp(16)1Yey/+* mice. *RCAN1* reduction improves calcineurin activity in *Dp(16)1Yey/+:RCAN*^*+/−*^ mice relative to *Dp(16)1Yey/+* litter-mates. Calcineurin phosphatase activity was measured using a colorimetric assay that detects free phosphate released from the calcineurin-specific RII phosphopeptide. Results are mean±s.e.m. from *n*=6 mice per genotype. **P<0.01, ***P<0.001. (**c**,**d**) Reducing *RCAN1* gene dosage restores dynamin1 phosphorylation status in *Dp(16)1Yey/+* mice. *Dp(16)1Yey/+* mice have increased levels of phospho-dynamin1 in sympathetic axons *in vivo*, that is corrected by removing one copy of *RCAN1* in *Dp(16)1Yey/+:RCAN*^*+/−*^ mice. Salivary gland lysates from P0.5 wild type, *Dp(16)1Yey/+* and *Dp(16)1Yey/+:RCAN*^*+/−*^ mice were immunoblotted using phospho-dynamin1 (Ser778) antibody. Immunoblots were stripped and reprobed for total dynamin1 for normalization. (**d**) Densitometric quantification of phospho-dynamin1 (Ser778) after treatments as described in **c**. Values are expressed relative to wild type. Results are means±s.e.m. from *n*=7 mice per genotype. *P<0.05, **P<0.01. (**e**–**j**) Reducing *RCAN1* gene dosage rescues defective Trk receptor endocytosis in *Dp(16)1Yey/+* mice. Scale bar, 5 μm and 10 μm for axons and cell bodies, respectively. (**k**,**l**) Quantification of internalized Trk in cell bodies and axons. 40–50 cells were analysed per condition per experiment. Quantification is represented as fold-change relative to wild-type neurons with no ligand. Results are the mean±s.e.m. from at least five independent experiments. NS, not significant, *P<0.05, **P<0.01, ***P<0.001. Statistical analyses done by one-way ANOVA and Tukey's *post-hoc* test for **a**,**b**,**d**, and two-way ANOVA and Bonferroni *post-hoc* test for **k**,**l**. Full-length blot scans are shown in Supplementary Fig. 8.

Our findings in compartmentalized sympathetic cultures suggest a mechanism by which excess RCAN1 contributes to the sympathetic defects in *Dp(16)1Yey/+* mouse model of Down syndrome by impairing NGF-dependent TrkA endocytosis and retrograde trophic signalling. If this is indeed the case, then trisomic expression of *RCAN1* should recapitulate the phenotypes in *Dp(16)1Yey/+* mice. Thus, we employed *RCAN1* transgenic mice that express three copies of *RCAN1.4*, generated by targeting a myc-tagged third copy of *RCAN1.4* cDNA driven by its native promoter into the *Hprt* locus^43^. These mice exhibit a ∼2.5- to 3-fold increase in *RCAN1* mRNA levels, similar to Down syndrome human fetal tissues^43^, and *Dp(16)1Yey/+* mice (see Fig. 7a). Similar to the 45% decrease in SCG neuronal numbers observed in *Dp(16)1Yey/+* mice, we found a significant loss of sympathetic neurons in *RCAN1* trisomic mice at birth (13,002±1,112 neurons in P0.5 *RCAN1* mice versus 27,463±1,774 in wild-type litter-mates; Fig. 6a–c), as well as enhanced apoptosis in SCGs (Fig. 6d–f). In addition, *RCAN1* transgenic mice had significantly reduced sympathetic fibres in target tissues including the olfactory epithelium (Fig. 6g–i) and salivary glands (Fig. 6j–l).

The onset of developmental deficits in *RCAN1* transgenic mice was remarkably similar to that observed in *Dp(16)1Yey/+* mice. At early stages, that is, E16.5, there were no obvious differences between *RCAN1* and wild-type mice in the formation of the SCG and sympathetic neuronal numbers (Supplementary Fig. 6a–c), indicating a selective effect on post-mitotic neurons that depend on target-derived NGF. Also, sympathetic innervation deficits preceded the neuronal loss in *RCAN1* trisomic embryos (Supplementary Fig. 6d–f), suggesting that RCAN1 accumulation in the two different *RCAN1* trisomic mouse models may have an early effect on attenuating axon growth at sympathetic nerve terminals.

In light of our observations that reduced neuronal numbers is manifested at birth well after the deficits in sympathetic innervation of target tissues in both *RCAN1* transgenic and *Dp(16)1Yey/+* embryonic mice, we conclude that the neuronal loss may be a cumulative effect that reflects the failure of sympathetic axons to gain access to adequate levels of target-derived NGF because of the diminished innervation, as well as reduced retrograde trafficking of TrkA receptors from nerve terminals to neuronal cell bodies. Together, these findings indicate that *RCAN1* triplication alone is sufficient to perturb NGF-dependent trophic functions during sympathetic nervous system development.

### *RCAN1* normalization improves Trk endocytosis in DS mice

To further test the causal role of *RCAN1* trisomy in defects in TrkA trafficking and NGF-dependent development, we eliminated one copy of *RCAN1* in *Dp(16)1Yey/+* mice by crossing these mice with *RCAN1*^*+/−*^ mice^48^ to generate *Dp(16)1Yey/+:RCAN*^*+/−*^ mice, which will have two copies of *RCAN1* but maintain trisomy of the other 112 genes. Quantitative real-time PCR analysis of SCGs confirmed that *RCAN1* levels were restored to physiological levels in *Dp(16)1Yey/+:RCAN*^*+/−*^ mice (Fig. 7a).

To assess the effects of an extra copy of *RCAN1* on restraining calcineurin signalling, we compared calcineurin phosphatase activity in SCG lysates from P0.5 wild-type mice with that in *Dp(16)1Yey/+* mice containing two versus three copies of *RCAN1*. Calcineurin activity was substantially decreased in SCG lysates from *Dp(16)1Yey/+* mice with three *RCAN1* copies (42% of wild-type activity), but reducing *RCAN1* gene dosage significantly improved calcineurin activity (64% of wild-type activity) in *Dp(16)1Yey/+:RCAN*^*+/−*^ mice (Fig. 7b). To test whether the improvement in calcineurin activity by removing one copy of *RCAN1* is reflected by altered dynamin phosphorylation, we assessed levels of phospho-dynamin1 in salivary glands *in vivo*. The salivary glands are richly innervated by axonal projections from the SCG, and immunoblotting of salivary gland lysates with the phospho-dynamin1 antibody should reveal the phosphorylation status of dynamin1, a neuron-specific protein, locally in nerve terminals. We found a significant increase (2.5±0.4-fold increase) in phosphorylated dynamin1 (P-Ser-778) in sympathetic axons innervating the salivary glands in *Dp(16)1Yey/+* mice compared with wild-type controls. Notably, this increase in phosphorylated dynamin1 was corrected by reducing *RCAN1* dosage in *Dp(16)1Yey/+:RCAN*^*+/−*^ mice (Fig. 7c,d). There were no significant differences in levels of phosphorylated dynamin1 between *Dp(16)1Yey/+:RCAN*^*+/−*^ mice and wild-type litter-mates. Together, these findings support a key contribution of the extra copy of *RCAN1* in suppressing calcineurin activity and increasing dynamin1 phosphorylation in *Dp(16)1Yey/+* sympathetic neurons.

Given that reducing *RCAN1* gene dosage ameliorated deficits in calcineurin activity and dynamin1 phosphorylation in *Dp(16)1Yey/+* mice, we then asked whether this rescue approach would also alleviate defects in TrkA trafficking in *Dp(16)1Yey/+* neurons. Thus, we monitored the internalization of Trk receptors in compartmentalized cultures of sympathetic neurons isolated from P0.5 SCGs of *Dp(16)1Yey/+*, litter-mate *Dp(16)1Yey/+:RCAN*^*+/−*^ and wild-type mice. We found that, in contrast to the internalization defect in *Dp(16)1Yey/+* neurons, neurotrophin stimulation resulted in significant Trk receptor internalization in *Dp(16)1Yey/+:RCAN*^*+/−*^ rescue neurons similar to that in wild-type neurons (Fig. 7e–j). The normalization of receptor endocytosis with reduced *RCAN1* gene dosage was evident in both cell bodies (Fig. 7k) and axons (Fig. 7l) from *Dp(16)1Yey/+:RCAN*^*+/−*^ sympathetic neurons. Together, these results establish a causal link between excess RCAN1 and defects in neurotrophin receptor trafficking in *Dp(16)1Yey/+* neurons.

### Reducing *RCAN1* dosage alleviates developmental phenotypes

**Figure 8:**
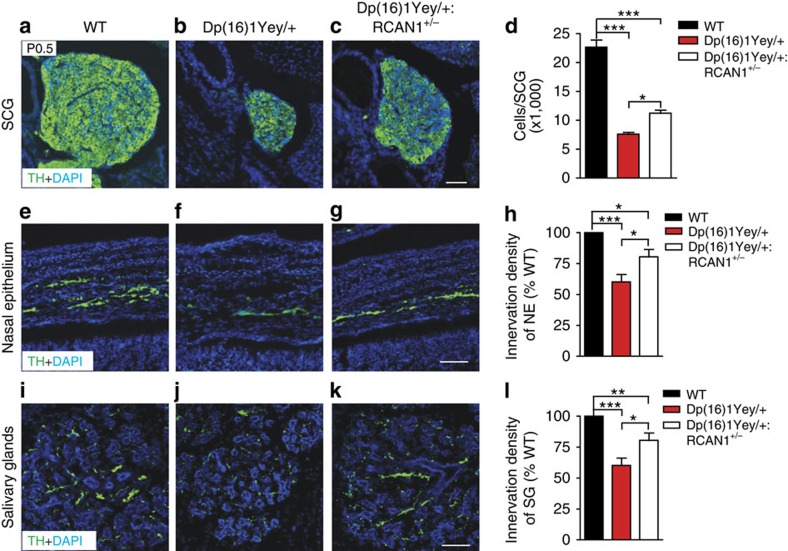
Reducing *RCAN1* gene dosage improves neuronal survival and target innervation in *Dp(16)1Yey/+* mice. (**a**–**d**) Genetic reduction of *RCAN1* ameliorates the loss of sympathetic neurons in *Dp(16)1Yey/+* mice. P0.5 SCGs from *Dp(16)1Yey/+:RCAN*^*+/−*^ mice show increased size and neuronal numbers compared with *Dp(16)1Yey/+* litter-mates. SCGs were visualized by TH immunohistochemistry and cell counts were performed on Nissl-stained tissue sections. Results are the mean±s.e.m. from *n*=5 mice per genotype. *P<0.05, ***P<0.001. (**e**–**l**) TH immunohistochemistry reveals a marked improvement of sympathetic innervation in target tissues in P0.5 *Dp(16)1Yey/+:RCAN*^*+/−*^ mice compared with *Dp(16)1Yey/+* litter-mates. Innervation of nasal epithelium is shown in **e**–**h** and that of salivary glands in **i**–**l**. Quantification of innervation density (**h**, nasal epithelium; **l**, salivary glands) was estimated from *n*=6 mice per genotype. Values are the mean±s.e.m. The results are represented as a percentage of the mean for wild-type mice. *P<0.05, **P<0.01, ***P<0.001. Scale bar, 100 μm for **a**,**b**,**c**,**i**,**j**,**k** and 50 μm for **e**,**f**,**g**. Statistical analyses by one-way ANOVA and Tukey's *post-hoc* test for **d**,**h**,**l**.

We then addressed whether reducing *RCAN1* levels would rescue the phenotypes in sympathetic nervous system development in *Dp(16)1Yey/+* mice. As expected, we observed a substantial decrease in SCG cell numbers in *Dp(16)1Yey/+* mice compared with wild-type pups (7,601±334 neurons in *Dp(16)1Yey/*^*+*^ mice versus 22,680±1,251 in wild-type litter-mates; Fig. 8a,b,d). Genetically reducing *RCAN1* levels ameliorated the loss of sympathetic neurons since we found a significant increase (32.4%) in SCG cell numbers in *Dp(16)1Yey/+:RCAN*^*+/−*^ mice compared with their *Dp(16)1Yey/+* litter-mates (11,259±556 neurons in *Dp(16)1Yey/+:RCAN*^*+/−*^ mice versus 7,601±334 neurons in *Dp(16)1Yey/*^*+*^ litter-mates; Fig. 8c,d). Notably, the reduction in *RCAN1* dosage did not fully rescue the neuronal number deficit. Examination of sympathetic innervation also revealed a beneficial effect of reducing *RCAN1* levels since more TH-positive sympathetic fibres were found within the nasal epithelium (Fig. 8e–h) and salivary glands (Fig. 8i–l) in *Dp(16)1Yey/+:RCAN*^*+/−*^ mice compared with their *Dp(16)1Yey/+* litter-mates.

Together, these findings indicate that increased gene dosage of *RCAN1* significantly contributes to disruptions in NGF-dependent development of sympathetic neurons in *Dp(16)1Yey/+* mice.

## Discussion

In this study, we report the aberrant development of the sympathetic nervous system in Down syndrome. This finding provides an anatomical basis for the reported autonomic dysfunction in the disease. Down syndrome is a complex genetic disorder believed to arise from the trisomy of many genes. Here, we define that increased dosage of a single gene, *RCAN1*, exerts pronounced effects on the development of sympathetic neurons. We further show that excess RCAN1, a genetic inhibitor of calcineurin phosphatase activity, interferes with phosphoregulation of dynamin1, internalization of TrkA receptors and retrograde trophic signalling by NGF (Supplementary Fig. 7). Collectively, our findings provide new insight into the poorly understood cellular pathways that are aberrant in Down syndrome, and define a framework for further investigations into the role of sympathetic innervation in the pathogenesis of the disease.

A key question is whether, in the context of trisomy of 113 genes in the *Dp(16)1Yey/+* mouse model of Down syndrome, increased expression of *RCAN1* is primarily responsible for the sympathetic nervous system phenotypes that we observed? We found that triplication of *RCAN1* alone was sufficient to mimic the loss of neurons and diminished sympathetic innervation in *Dp(16)1Yey/+* mice. We also showed that deleting a single copy of *RCAN1* in the *Dp(16)1Yey*/+ mouse model led to marked improvements in the sympathetic nervous system phenotypes, although reducing *RCAN1* dosage did not fully restore neuronal numbers and innervation in *Dp(16)1Yey*/+ mice. Interestingly, calcineurin activity, although significantly improved by reducing *RCAN1* levels in *Dp(16)1Yey*/+ mice, was also not completely restored. Thus, in *Dp(16)1Yey*/+ mice, decreased calcineurin activity may not simply be attributable to increased RCAN1 expression but may also result from an imbalance of RCAN1 activity, perhaps mediated by interactions with other trisomic gene products. RCAN1 phosphorylation by dual specificity tyrosine phosphorylation-regulated kinase 1A (Dyrk1a), a trisomic gene product also known to regulate calcineurin signalling and implicated in Down syndrome phenotypes, has been reported to augment RCAN1's inhibitory activity towards calcineurin^49^. Therefore, increased gene dosage of *Dyrk1a* could act together with RCAN1 overexpression to suppress calcineurin activity and contribute to the observed sympathetic nervous system phenotypes in *Dp(16)1Yey*/+ mice. Our results, therefore, do not preclude the contribution of other trisomic genes to the disruptions in sympathetic nervous system development in *Dp(16)1Yey/+* mice, either by impinging on RCAN1 regulation as exemplified above for *Dyrk1a*, or via RCAN1-independent mechanisms. Nevertheless, based on our findings that *RCAN1* gene triplication alone is sufficient to substantially disrupt NGF-dependent development of sympathetic neurons, and importantly, that these defects in *Dp(16)1Yey/+* mice are significantly ameliorated by normalizing *RCAN1* levels, we conclude that an imbalance in *RCAN1* gene dosage is a key contributing mechanism to the sympathetic phenotypes in Down syndrome mice.

Our findings support the view that sympathetic defects in *Dp(16)1Yey/+* mice arise from a failure in target-derived NGF signalling. In *Dp(16)1Yey/+* mice, we observed attenuated innervation of end-organs and loss of post-mitotic neurons during a developmental period (E16.5 to P0.5) of known dependence on NGF trophic signalling^16^. The sympathetic deficits in *Dp(16)1Yey/+* mice are reminiscent of phenotypes observed in mice lacking *NGF* or *TrkA*^17^,^18^,^19^,^29^. Although several cellular processes could potentially contribute to enhanced apoptosis and diminished innervation in *Dp(16)1Yey/+* mice, our findings support excess RCAN1-mediated disruption of TrkA endocytosis as a key mechanism. The retrograde accumulation of NGF protein was decreased in *Dp(16)1Yey/+* sympathetic ganglia, despite normal NGF production in targets. TrkA receptor endocytosis in nerve terminals is an essential step in retrograde NGF trophic signalling^23^,^29^,^47^. Indeed, ligand-dependent TrkA endocytosis was abolished in *Dp(16)1Yey/+* and RCAN1 overexpressing sympathetic neurons, whereas deleting a copy of *RCAN1* in *Dp(16)1Yey/+* sympathetic neurons normalized receptor internalization, which correlated with improved neuronal survival and target innervation *in vivo*. Furthermore, RCAN1 overexpression in compartmentalized neurons markedly compromised the ability of NGF to support retrograde trophic signalling. Together, these findings provide evidence in support of a relationship between excess RCAN1, impaired TrkA endocytosis and decreased neurotrophic support of NGF-responsive sympathetic neurons in *Dp(16)1Yey/+* mice. During endocytic trafficking in neurons, neurotrophin receptors are not passive passengers being carried along by a constitutively operating endocytic machinery. Rather, Trk receptor tyrosine kinase-initiated signals actively modulate the endocytic machinery to drive their own trafficking^31^. We previously demonstrated that TrkA signalling drives its own internalization by recruiting PLC-γ, which then stimulates calcineurin-dependent dephosphorylation of neuron-specific splicing isoforms of dynamin1 (ref. 34). Here, we show that excess RCAN1 interferes with this TrkA-initiated endocytic signalling pathway, but does not generally impede clathrin-mediated endocytosis. Furthermore, we found that RCAN1 overexpression uniquely compromises neuronal survival when NGF is present on distal axons, but that RCAN1 overexpressing neurons are healthy and viable with NGF added directly to cell bodies. Together, these findings support the view that excess RCAN1 does not elicit non-specific endocytic deficits in sympathetic neurons, but interferes with the propagation of long-distance NGF endosomal signalling.

Intriguingly, similar to Down syndrome individuals, *RCAN1* mRNA levels are elevated two- to threefold in the brains of Alzheimer's disease patients^50^. Almost all individuals with Down syndrome exhibit early-onset neurodegeneration with pathological features similar to Alzheimer's disease and many familial cases of Alzheimer's are linked to human chromosome 21 genes, indicative of common pathogenetic mechanisms^51^,^52^,^53^. These findings raise a question of whether RCAN1 accumulation could underlie decreased neurotrophic support and ultimate degeneration of NGF-responsive adult CNS neurons. Basal forebrain cholinergic neurons are a TrkA-expressing CNS population that undergo age-dependent atrophy in humans with Down's syndrome and Alzheimer's disease^54^,^55^,^56^. As mouse models of Down syndrome and Alzheimer's disease exhibit decreased retrograde transport of NGF from hippocampal and cortical target regions of basal forebrain cholinergic neurons, disturbed trophic support has been suggested to underlie neuronal atrophy^24^,^57^,^58^. In contrast to the well-established trophic dependence of developing peripheral neurons on NGF:TrkA signalling and trafficking, genetic deletion of *NGF* or *TrkA* in mice does not compromise the survival of the majority of basal forebrain cholinergic neurons^17^,^59^,^60^,^61^,^62^, but results in defects in cholinergic input to the cortex and hippocampus and the downregulation of cholinergic neurotransmitter synthesizing enzymes^59^,^60^,^61^. When tested in behavioral tasks, conditional mutant mice lacking NGF:TrkA signalling showed either no cognitive impairment^62^ or mild cognitive decline^61^. Unlike sympathetic neurons that rely on a single neurotrophin, NGF, for neuronal survival and target innervation during development, basal forebrain cholinergic neurons are responsive to multiple neurotrophins^63^. In addition, although the requirement of TrkA trafficking in promoting sympathetic neuron development is well-documented^31^,^32^, there is less evidence to support a role for TrkA trafficking in basal forebrain cholinergic neuron development and maturation. Thus, further studies are warranted to clarify the relationship between TrkA trafficking and neurotrophic support of basal forebrain cholinergic neurons, and to define a possible contribution of excess RCAN1 to neurodegeneration.

Previously, we found that genetic ablation of sympathetic innervation during early development resulted in altered pancreatic islet architecture and functional deficits in insulin secretion and glucose metabolism in mice^64^. Several peripheral organs are abundantly innervated by sympathetic fibres, initiated during development, yet, the role of innervation as a contributing mechanism to perturbations in organogenesis and dysfunction in Down syndrome has received little attention. Delayed or aberrant development of peripheral organs such as the heart, thymus and gastrointestinal tract are manifested in Down syndrome^11^,^65^. Our current findings provide a platform for further investigations to determine whether developmental deficits in autonomic innervation underlie the well-established impairments in cardiovascular, endocrine and immune functions in Down syndrome.

## Methods

### Human tissues

Human Down syndrome peripheral tissues were obtained from the NIH NeuroBioBank. Details of the specimens are provided in Supplementary Table 1. Research with human-derived materials was registered with the Johns Hopkins Biosafety Office and was performed in compliance with all institutional and governmental guidelines.

### Animals

All procedures relating to animal care and treatment conformed to Johns Hopkins University Animal Care and Use Committee and NIH guidelines. Animals were housed in a standard 12:12 light–dark cycle. Mice were maintained on a *C57BL/6* background, or mixed *C57BL/6* and *Balb/C* background in the case where *Dp(16)1Yey* mice were crossed to *RCAN1* heterozygous mice. Both sexes were used for analyses. Trisomic *RCAN1* and *RCAN1*^*+/−*^ mice were a generous gift from Dr Sandra Ryeom. *RCAN1* trisomic mice were genotyped by PCR screening to ensure that only heterozygous mice were used for *in vivo* analyses of *RCAN1* trisomy^43^. Generation and genotyping of *Dp(16)1Yey/+* mice have been described previously^26^, and the mice were generously provided by Dr Eugene Yu and Dr Roger Reeves. *Dp(16)1Yey/+;RCAN*^*+/−*^ mice that were diploid for *RCAN1* and trisomic for the rest of human chromosome 21 syntenic region on mouse chromosome 16 were generated by mating *RCAN1*^*+/−*^ mice with *Dp(16)1Yey/+* mice, and litters were genotyped as previously described^26^,^43^.

Sprague–Dawley rats were purchased from Charles River. Sympathetic neuron cultures were established from superior cervical ganglia dissected from P0.5 rat pups as previously described^66^.

### Constructs, reagents and antibodies

Human HA-tagged RCAN1.4, a gift from Dr Kyle Cunningham, was sub-cloned into pShuttle-CMV vector (Stratagene) and a replication incompetent recombinant adenoviral construct was generated using the AdEasy adenoviral vector system (Stratagene). Recombinant viral backbone was transfected into HEK 293 cells (American Type Culture Collection) using Lipofectamine (Invitrogen) and high-titre virus stocks purified using a CsCl gradient. The generation of the FLAG-TrkB/A adenovirus has been previously described^33^. The antibodies used in this study were previously validated for the following applications: P-Tyr (Sigma-Aldrich; P4110, immunoprecipitation), P-Erk1/2 (Cell Signaling; 9106, western blotting), P-Akt (Cell Signaling; 9271, western blotting), TrkA (Millipore; AB1577, western blotting), p85 subunit of phosphatidylinositol-3-kinase (Upstate Biotechnology, 06–195, western blotting), dynamin1 (Abcam; ab3456, western blotting), dynamin phospho-Ser778 (Imgenex, IMG-670, western blotting), β-III-tubulin (Sigma-Aldrich; T8578, immunocytochemistry), HA (Sigma-Aldrich; H9658, western blotting), transferrin (Santa Cruz Biotechnology; sc-52256, western blotting), FLAG M2 (Sigma-Aldrich; H9658, antibody feeding assays), TH (Millipore; AB152, immunohistochemistry), cleaved caspase-3 (Cell Signaling; 9661, immunohistochemistry), NF200 (Sigma-Aldrich; 4142, immunohistochemistry), insulin (Dako; A0564, immunohistochemistry), glucagon (Abcam; ab10988, immunohistochemistry) and α-smooth muscle actin-FITC (Sigma-Aldrich; F3777, immunohistochemistry). The RCAN1 antibody was generated by injecting rabbits with the peptide FLISPPASPPV, and blood serum was extracted (Pocono Rabbit Farm). RCAN1 anti-serum was used directly at a dilution of 1:1,000 for immunoblotting and 1:200 for immunocytochemistry. FK-506 and CsA were obtained from Sigma-Aldrich. Calcineurin activity was assessed using the Enzo cellular activity assay kit (AK816, Enzo Life Sciences) and substrate reaction was measured using a plate reader. NGF levels were determined by ChemiKine NGF Sandwich ELISA kit (cat #: CYT304) from Millipore. TH levels were determined by TH Sandwich ELISA Kit from Biomatik (cat #: EKU08003).

### Histological analyses of human tissues

Human tissues were fixed at 4 °C for 3–4 h in 4% paraformaldehyde (PFA), incubated in 30% sucrose overnight and embedded in optimal cutting temperature (OCT, Tissue-Tek). Tissue sections (20 μm) were washed with PBS, blocked in 5% BSA and 0.3% Triton X-100 for 1 h. Primary antibody incubation (TH, NF200, insulin, glucagon, α-smooth muscle actin-FITC) was performed overnight at room temperature in Can Get Signal enhancer solution from Toyobo. All primary antibodies were used at 1:200 dilution. Following incubation with secondary antibodies (1:200, 2 h, room temperature) and washes, slides were mounted with Prolong Antifade and 4,6-diamidino-2-phenylindole (DAPI). Images were acquired by confocal microscopy as three-dimensional reconstructions from *z*-stacks. Quantification of innervation density in spleen and pancreatic tissues was done by measuring the integrated density of fluorescent pixels for TH or neurofilament immunostaining (ImageJ). Density was normalized to unit area, and at least 50 images per tissue were averaged. Results were expressed as arbitrary fluorescence units per 100 μm^2^.

Haematoxylin and eosin staining was performed by the Johns Hopkins Oncology Histology Laboratory. The histological, imaging and quantification analyses were performed such that the investigator was blinded to the group allocations.

### Immunohistochemical analyses of mouse tissues

Mice at various developmental ages were fixed in 4% PFA at 4 °C for 3–4 h, cryoprotected in 30% sucrose in PBS, frozen in OCT and serially sectioned (12 μm). For immunofluorescence, sections were washed in PBS, permeabilized in PBS containing 1% Triton X-100 and blocked using 5% goat serum in PBS+0.1% Triton X-100. Sections were incubated in the following primary antibodies overnight: rabbit anti-TH (1:200; Millipore) and rabbit anti-cleaved caspase 3 (1:200; Cell Signaling). Following PBS washes, sections were incubated with anti-rabbit Alexa-488 secondary antibodies (1:200; Life Technologies). Sections were then washed in PBS and mounted in VectaShield (Vector Laboratories) containing 100 μg per ml DAPI.

Quantification of sympathetic innervation density in the salivary glands and nasal epithelium was done by calculating integrated TH fluorescence density per unit area (ImageJ) from multiple random images.

### Whole-mount TH immunohistochemistry

Whole-mount TH immunohistochemistry was performed as previously described^29^. Briefly, whole E16.5 mouse embryos were subjected to diaminobenzidine-TH immunohistochemistry, using a rabbit anti-TH (Millipore, AB152) at 0.5 μg ml^−1^ incubated for 72 h at 4 °C. Detection was performed with horseradish peroxidase-conjugated donkey anti-rabbit IgG (GE Healthcare) at 4 μg ml^−1^ incubated overnight at 4 °C. Visualization was accomplished with diaminobenzidine (Sigma-Aldrich), followed by clearing in 2:1 benzyl benzoate/benzyl alcohol (Sigma-Aldrich).

### *In situ* hybridization

*In situ* hybridization was performed using a digoxigenin-labelled probe spanning a 480-bp region within exons 5–7 of mouse *RCAN1*. P0.5 mouse pups were fresh frozen in OCT and serially sectioned (12 μm) using a cryostat. Sections were post-fixed in 4% PFA, washed in PBS and acetylated with 0.25% acetic anhydride in 0.1 M triethanolamine with 0.9% NaCl. After hybridization with the labelled RNA probe (2 μg per ml) at 68 °C overnight, sections were washed with 0.2 × SSC buffer at 65 °C, blocked with TBS containing 1% normal goat serum and then incubated with alkaline phosphatase-labelled anti-DIG antibody (1:5,000; Roche) overnight at 4 °C. The alkaline phosphatase reaction was visualized with NBT/BCIP, rinsed in PBS, fixed in 4% PFA and mounted in AquaMount (EMD Chemicals).

### RT–PCR analyses

Total RNA was prepared from dissected SCGs using Trizol-choloroform extraction (Life Technologies). RNA was then reverse transcribed using a RETROscript kit (Ambion). Real-time qPCR was performed using a Maxima SYBR Green/Rox Q-PCR Master Mix (Thermo Scientific), in a 7300 Real-time PCR System (Applied Biosystems). *RCAN1* mRNA levels were measured by using primers targeting exons 5–7 (*RCAN1*-F: 5′- TTCCTGGGGAAGGAAATGAA -3′ and *RCAN1*-R: 5′- GGTGGTGTCCTTGTCATATG -3′). Glyceraldehyde-3-phosphate dehydrogenase (GAPDH) was used as a control (*GAPDH*-F: 5′- CCTGCACCACCAACTGCTTA -3′ and *GAPDH*-R: 5′- CCACGATGCCAAAGTTGTCA -3′). Each sample was analysed in triplicate reactions. Fold change in *RCAN1* transcript levels was calculated using the 2^(−ΔΔCt)^ method, normalizing to GAPDH transcript.

RT–PCR analyses for *RCAN1.1* and *RCAN1.4* isoforms were performed using the following primers: *RCAN1.1*-F 5′- ACTGGAGCTTCATCGACTGC -3′ and *RCAN1.4*-F: 5′- AGCTCCCTGATTGCTTGTGT -3′ and a common reverse primer 5′- GTGTACTCCGGTCTCCGTGT -3′.

### NGF and TH ELISA assays

NGF levels were assessed using a NGF Sandwich ELISA Kit (Millipore). Briefly, SCGs, salivary glands and hearts were dissected from P1 mouse pups, homogenized and centrifuged. Salivary gland and heart supernatants were diluted, and all tissue lysates were incubated in ChemKine wells overnight at 4 °C. With the ChemiKine NGF assay system, sheep polyclonal antibodies generated against mouse NGF are coated onto a microplate and are used to capture NGF from a sample. After washing, samples were incubated with NGF-specific mouse monoclonal antibodies (1:100 dilution) for 2 h at room temperature to detect the captured NGF, followed by incubation with peroxidase-conjugated secondary antibody (2 h, room temperature), and TMB/E substrate (5 min, room temperature). The reaction was stopped and development was assessed using a plate reader.

TH levels were assessed using a TH Sandwich ELISA Kit (Biomatik EKU08003). SCGs dissected from P1 mice were homogenized and centrifuged. Lysates were incubated in antibody-coated wells for 3 h at 37 °C. After washing, samples were incubated with a biotin-conjugated antibody specific to TH for 1 h at 37 °C, followed by incubation with avidin conjugated to horseradish peroxidase for 30 min at 37 °C, TMB/E substrate for 15 min at 37 °C, and assessed using a plate reader.

NGF protein levels were normalized to TH protein levels in SCGs, and to total protein concentrations as determined by a bicinchoninic acid (BCA) protein assay in salivary gland and heart lysates.

### Calcineurin activity assay

SCGs, salivary glands and heart tissues were dissected from P0.5 mouse pups and homogenized in lysis buffer containing protease inhibitors. Supernatants were incubated with the calcineurin-specific substrate, the RII phosphopeptide, at 37 °C for 30 min. Phosphatase activity was measured by colorimetric detection of free phosphate using a plate reader. Total phosphatase activity was assessed under conditions where no exogenous substrate was included, in a calcineurin-permissive buffer (inclusion of calmodulin) and a calcineurin-inhibiting buffer (inclusion of the calcium chelator, EGTA). Calcineurin phosphatase activity was calculated by subtracting calcineurin-independent phosphatase activity (in the EGTA condition) from total phosphatase activity. Phosphatase activity was normalized to total protein using a BCA protein assay.

### Neuronal cell counts

E16.5 and P0.5 mice for neuronal counts were prepared as described^67^. Briefly, mouse torsos were fixed in PBS containing 4% PFA, and then cryoprotected overnight in 30% sucrose-PBS. SCG sections (12 μm) were stained with a solution containing 0.5% cresyl violet (Nissl). Cells with characteristic neuronal morphology and visible nucleoli were counted in every fifth Nissl-stained section.

### Neuronal cultures and adenovirus infection

Sympathetic neurons were harvested from P0.5 Sprague–Dawley rats and grown in mass cultures or compartmentalized cultures as described previously^29^. Dissociated DRG neurons were isolated from E15–16 rats and grown in mass cultures using culture conditions similar to that described for sympathetic neurons. Cells were maintained in culture with high-glucose DMEM media supplemented with 10% fetal bovine serum, penicillin/streptomycin (1 U perml) and NGF (100 ng perml) purified from male mouse submaxillary glands as described previously^68^. For immunocytochemistry, cells were plated on poly-D-lysine-coated (1 μg perml; Sigma-Aldrich) coverslips. To withdraw NGF before any stimulation experiments, neurons were placed in high-glucose DMEM containing 0.5% fetal bovine serum with 1:1,000 anti-NGF (Sigma-Aldrich) and BAF (50 μM; MP Biomedical) for 48 h. For adenoviral infections, neuronal cultures were infected with high-titre CsCl-purified adenoviruses for 48 h as described previously^69^.

### Neuronal survival and axon growth assays

Sympathetic neurons grown in compartmentalized cultures were infected with adenoviral constructs expressing LacZ or RCAN1. To ensure survival scoring of only the neurons that had projected axons into the side chambers, fluorescent microspheres (Invitrogen) were added to the distal axon compartments 24 h before the experiments. Neurons were either completely deprived of NGF (by adding anti-NGF at 1:1,000 dilution to both cell body and distal axon compartments) or supported by NGF (100 ng per ml) added only to distal axons or cell bodies. After 72 h, neurons were fixed and dying cells were visualized using TUNEL staining (Roche) according to the manufacturer's protocol. Neuronal apoptosis was calculated by determining the percentage of neurons that had extended axons into the side chambers (visualized by fluorescent microsphere uptake) that were also positive for TUNEL label.

For assessing axon growth, compartmentalized neuronal cultures were infected with GFP or RCAN1. Neurons were either completely deprived of NGF or NGF (100 ng per ml) was added only to distal axons as for the survival assays. The broad-spectrum caspase inhibitor, BAF (50 μM) was also included to allow assesement of axon growth without the complications of cell death. Axon growth was quantified by capturing phase contrast images of the distal axon compartments over consecutive 24 h intervals for 3 days, using a Zeiss Axiovert 200 microscope with a Retiga EXi camera. Rate of axonal growth (μm per day) was measured using Openlab 4.04. Measurements from 30–50 neurons were averaged for each condition for a single experiment. For representative images, neurons were immunostained with β-III-tubulin (1:200; Sigma-Aldrich) 24 h after the NGF treatment.

### TrkA receptor internalization assays

Cell surface biotinylation assays were performed in cultured rat sympathetic neurons as previously described^29^. Briefly, neurons were biotinylated with a reversible membrane-impermeable form of biotin (EZ-Link NHS-S-S-biotin, 1.5 mg per ml in PBS; Pierce Chemical) at 4 °C for 25 min. Neurons were washed briefly with PBS containing 50 mM glycine (Sigma-Aldrich) to remove remaining unconjugated biotin. Neurons were moved to 37 °C to promote internalization under the appropriate conditions for 30 min. Neurons were returned to 4 °C and the remaining biotinylated surface receptors were stripped of their biotin tag with 50 mM glutathione (Sigma-Aldrich). After this stripping process, cells were washed twice with 50 mM iodoacetamide (Sigma-Aldrich) to quench excess glutathione. Neurons were lysed with 500 μl of RIPA buffer (50 mM Tris-HCl, 150 mM NaCl, 1 mM EDTA, 1% NP-40, 0.25% deoxycholate), and supernatants were subjected to precipitation with 40 μl immobilized neutravidin agarose beads (Pierce Chemical) and immunoblotting with a TrkA antibody.

Live-cell antibody feeding assays to monitor Trk receptor internalization were performed as previously described^33^. Briefly, sympathetic neurons harvested from P0.5 wild-type, *Dp(16)1Yey/+* and *Dp(16)1Yey/+;RCAN*^*+/−*^ mice were grown in microfluidic chambers for 2–4 days *in vitro* (d.i.v.) to allow axons to project into the outer chambers. Neurons were infected with an adenoviral vector that expresses GFP and FLAG-TrkB:A chimeric receptors. Infected neurons were identified by GFP expression. Forty-eight hours48 h post-infection, cultures were washed to remove all NGF, and incubated with mouse anti-FLAG antibody (M2, 4.2 μg per ml Sigma-Aldrich) for 30 min at 4 °C in PBS. Excess antibody was washed off followed by incubation with either control medium or medium containing BDNF (100 ng per ml) and the cells moved to 37 °C for 30 min to allow for internalization. Cells were then washed quickly with PBS and immediately fixed with 4% paraformaldehye in PBS for 30 min at room temperature. Cells were then permeabilized with 0.1% Triton X-100/1% BSA/PBS and incubated with fluorescently conjugated anti-mouse secondary antibody for 1 h, and then mounted on slides with Aquamount (Invitrogen). Images representing 0.8 μm optical slices were acquired using a Zeiss LSM 510 confocal scanning microscope equipped with Ar (458–488 nm) and He/Ne (543–633) lasers. The same confocal acquisition settings were applied to all images taken from a single experiment. Cell bodies were analysed by taking z-stacks through the entire cell, and creating a three-dimensional reconstruction using Image J. Axons were analysed using single images with an aperture of 0.8 μm. Threshold settings for green and red scans were determined, and the integrated fluorescence values for each channel were quantified. Internalization was quantified as the ratio of anti-FLAG immunofluorescence (red) that co-localized with cytoplasmic GFP (green) relative to the total anti-FLAG immunofluorescence. Weighted coefficients of co-localization between the anti-FLAG and GFP fluorescence were determined by Image J software.

A modified version of the antibody feeding assay was employed in experiments in Fig. 7e–j, where neurons, labelled with the FLAG antibody under non-permeabilizing conditions and stimulated with BDNF to allow ligand-mediated internalization, were then quickly stripped of surface-bound FLAG antibodies that had not internalized by three quick washes in an ice-cold acidic buffer (0.2 N acetic acid, 0.5 M NaCl, pH 3.0). Internalized receptors were then visualized by fixing, permeabilizing and incubation with fluorescently conjugated anti-mouse secondary antibody. Images were acquired using confocal microscopy and creating a three-dimensional model from *z*-stacks. Internalized receptors were calculated as Alexa-546 fluorescent pixels per μm^2^ of cell body or axon (for axons, measurements were taken from a stretch of axon equal to 100–250 μm length). For all imaging, 40–50 cells were analysed per condition per experiment.

### Transferrin and EGF internalization assays

Sympathetic neurons, infected with RCAN1 or GFP adenoviruses, were serum-starved and incubated with anti-NGF in the presence of BAF (50 μM) for 2 days before the internalization assay. For the biochemical transferrin uptake assay, neurons were incubated with biotin-labelled transferrin in PBS (25 μg per ml) at 4 °C for 30 min. Cells were then washed two times with PBS and either left at 4 °C or moved to 37 °C for 5 min to allow transferrin internalization. To measure internalized transferrin, surface-bound transferrin was stripped by adding 2 ml of ice-cold acidic buffer to the cells for 2 min, followed by a wash with 10 ml of ice-cold PBS. The stripping and washing steps were performed twice. After the stripping process, neurons were lysed with 500 μl of RIPA buffer, and supernatants were subjected to precipitation with 40 μl-immobilized neutravidin agarose beads (Pierce Chemical) and immunoblotting with a transferrin antibody (1:1,000; Santa Cruz Biotechnology).

For the fluorescent-based uptake assays, neurons were incubated with Alexa-555-labelled transferrin (25 μg per ml) or Alexa-555-labelled EGF in PBS (2 μg per ml) at 4 °C for 30 min. Neurons were then either left at 4 °C or moved to 37 °C for 5 min or 15 min, respectively, followed by washes with ice-cold PBS, fixation with 4% PFA and then mounted on slides with Fluoromount (Vectashield) and DAPI. Levels of labelled transferrin that accumulated intracellularly were assessed within 5 min to ensure that we were monitoring transferrin internalization in the absence of significant recycling back to the ;plasma membrane. Images were acquired using confocal microscopy and creating a three-dimensional model from *z*-stacks. Internalized receptors were calculated as Alexa-555 fluorescent pixels per μm^2^ of cell body. 40–50 cells were analysed per condition per experiment.

### Immunoblotting and immunoprecipitation assays

For biochemical analyses of local and retrograde NGF signalling, sympathetic neurons were grown in compartmentalized chambers for 7–10 d.i.v. until robust axonal projections were evident in the side compartments. Neurons were then infected with control GFP or RCAN1 adenoviruses and deprived of NGF for 48 h by adding anti-NGF in the presence of BAF (50 μM). Neurons were then either stimulated with NGF (100 ng per ml) added exclusively to distal axons for 8 h or treated with control medium alone, after which lysates were prepared separately from the cell body and distal axon compartments using RIPA buffer. To detect P-TrkA, lysates were subjected to immunoprecipitation with anti-phosphotyrosine (PY-20; Sigma-Aldrich) and incubated with Protein-A agarose beads (Santa Cruz Biotechnology) and immunoprecipitates were then immunoblotted for TrkA. The supernatants from the immunoprecipitations were subjected to immunoblotting for P-Akt, and P-Erk1/2. Normalization for protein amounts was done by immunoblotting for p85 (Upstate Biotechnology). In experiments to assess the role of local calcineurin activity on NGF signalling, distal axons of compartmentalized neurons were pre-treated with the calcineurin inhibitors, CsA (2 μg per ml) and FK506 (0.2 μg per ml) for 30 min before the NGF stimulation.

To detect phosphorylated dynamin1 *in vivo*, salivary glands were dissected from P1 mouse pups. To examine the phosphorylation level of dynamin1 *in vitro*, sympathetic neurons grown in mass cultures were stimulated with NGF for 30 min or treated with control medium. Lysates were prepared by laemmli sample buffer (62.5 mM Tris-HCl, 2% SDS, 10% Glycerol, 5% 2-mercaptoethanol, 0.01% bromophenol blue) and subjected to immunoblotting with the phospho-dynamin1 (P-Ser 778, 1:5,000; Imgenex) and dynamin1 (1:1,000; Abcam) antibodies. All immunoblots were visualized with ECL Plus Detection Reagent (GE Healthcare) and scanned with a Typhoon 9410 Variable Mode Imager (GE Healthcare).

### Statistical analyses

Samples sizes were similar to those reported in the previous publications^29^,^33^,^34^. Data were collected randomly and the assessment of human tissues was done in a manner blinded to the group allocation. For practical reasons, analyses of innervation in mouse tissues were done in a semi-blinded manner such that the investigator was aware of the genotypes before the experiment, but conducted the immunostaining and data analyses without knowing the genotypes of each sample. InStat software was used for statistical analyses. All Student's *t*-tests were performed assuming Gaussian distribution, two-tailed, unpaired and a confidence interval of 95%. For peripheral innervation of human tissue analyses, we used a one-tailed *t*-test, based on the prediction of directionality obtained from analyses in mice. One-way or two-way analyses of variance were performed when more than two groups were compared. Statistical analyses were based on at least three independent experiments, and described in the figure legends. All error bars represent the standard error of the mean (s.e.m.).

## Additional information

**How to cite this article:** Patel, A. *et al*. RCAN1 links impaired neurotrophin trafficking to aberrant development of the sympathetic nervous system in Down syndrome. *Nat. Commun.* 6:10119 doi: 10.1038/ncomms10119 (2015).

## Supplementary Material

Supplementary Information Supplementary Figures 1-8 and Supplementary Table 1
