# Supplementary material for: RCAN1 links impaired neurotrophin trafficking to aberrant development of the sympathetic nervous system in Down syndrome
Source: Nat Commun. 2015 Dec 14;6:10119. doi: 10.1038/ncomms10119 (PMC4682116; doi:10.1038/ncomms10119)
Supplement: Supplementary Information — Supplementary Figures 1-8 and Supplementary Table 1 [file ncomms10119-s1.pdf]

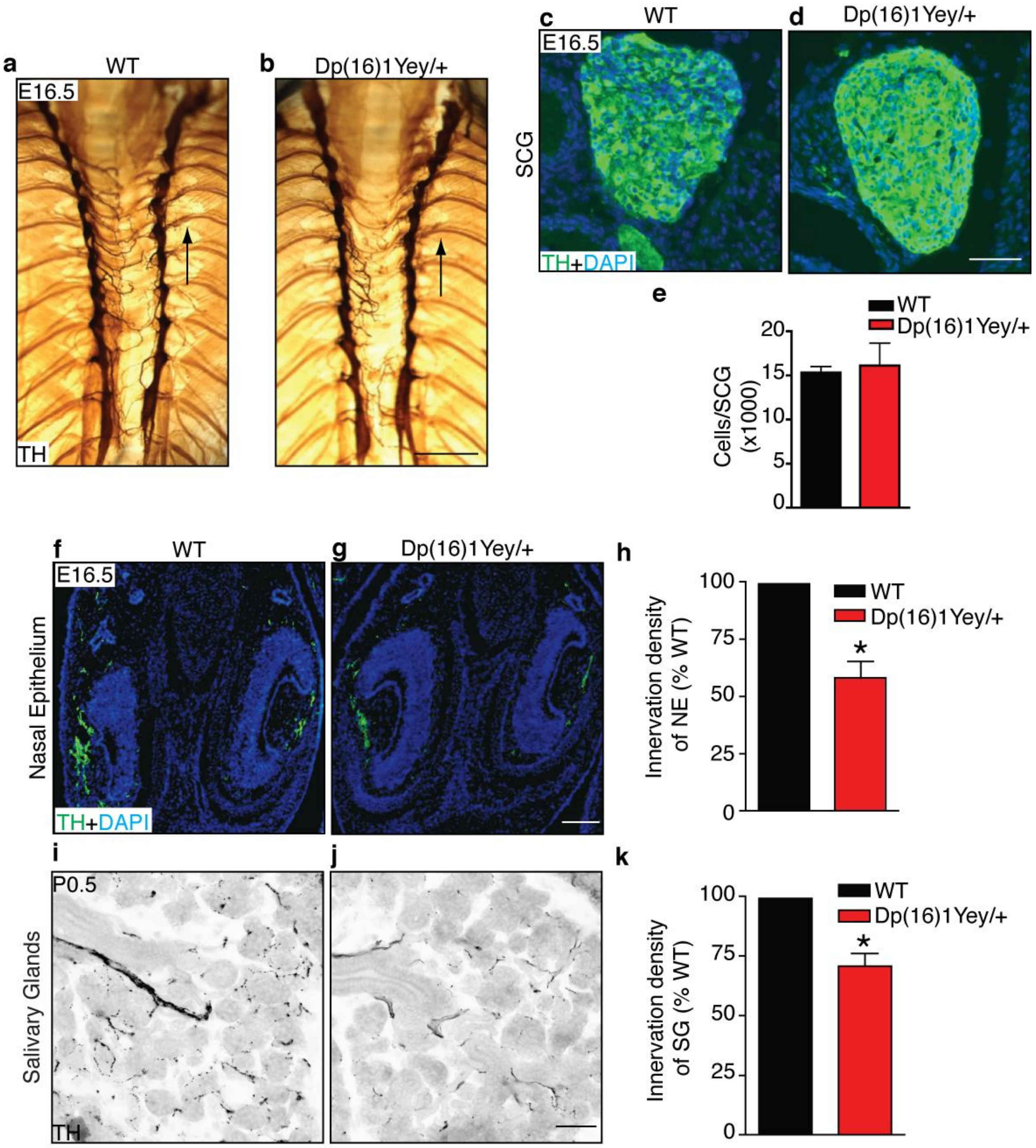

a
WT
E16.5
TH
b
Dp(16)1Yey/+
c
WT
E16.5
SCG
TH+DAPI
d
Dp(16)1Yey/+
e
WT
Dp(16)1Yey/+
Cells/SCG (x1000)
20
15
10
5
0
f
WT
E16.5
Nasal Epithelium
TH+DAPI
g
Dp(16)1Yey/+
h
Innervation density of NE (% WT)
100
75
50
25
0
WT
Dp(16)1Yey/+
*
i
P0.5
Salivary Glands
TH
j
k
Innervation density of SG (% WT)
100
75
50
25
0
WT
Dp(16)1Yey/+
*

Supplementary Figure 1. Analyses of sympathetic nervous system development in *Dp(16)1Yey/+* mice

(a,b) Whole-mount tyrosine hydroxylase immunostaining shows normal segmented organization of sympathetic chain ganglia and TH expression in *Dp(16)1Yey/+* mice at E16.5. Arrows indicate proximal projections from the sympathetic ganglia. Scale bar: 1 mm. **(c-e)** SCG morphology and cell numbers are normal in E16.5 *Dp(16)1Yey/+* mice, as well. SCGs were visualized by TH immunohistochemistry and cell counts were performed on Nissl stained tissue sections. Results are means ± SEM from n=3 mice per genotype. Scale bar: 100 µm. **(f-h)** However, sympathetic innervation of a target tissue, the nasal epithelium, is significantly reduced in E16.5 *Dp(16)1Yey/+* embryos. Scale bar: 100 µm. **(i-k)** Reduced sympathetic innervation of salivary glands in *Dp(16)1Yey/+* mice compared to wild-type litter-mates at birth (P0.5). Scale bar: 100 µm. Innervation density in the nasal epithelium and salivary glands was quantified from n=3 mice per genotype, and expressed as a percentage of wild-type values. Results are the mean ± SEM. *p<0.05 using unpaired two-tailed *t* test.

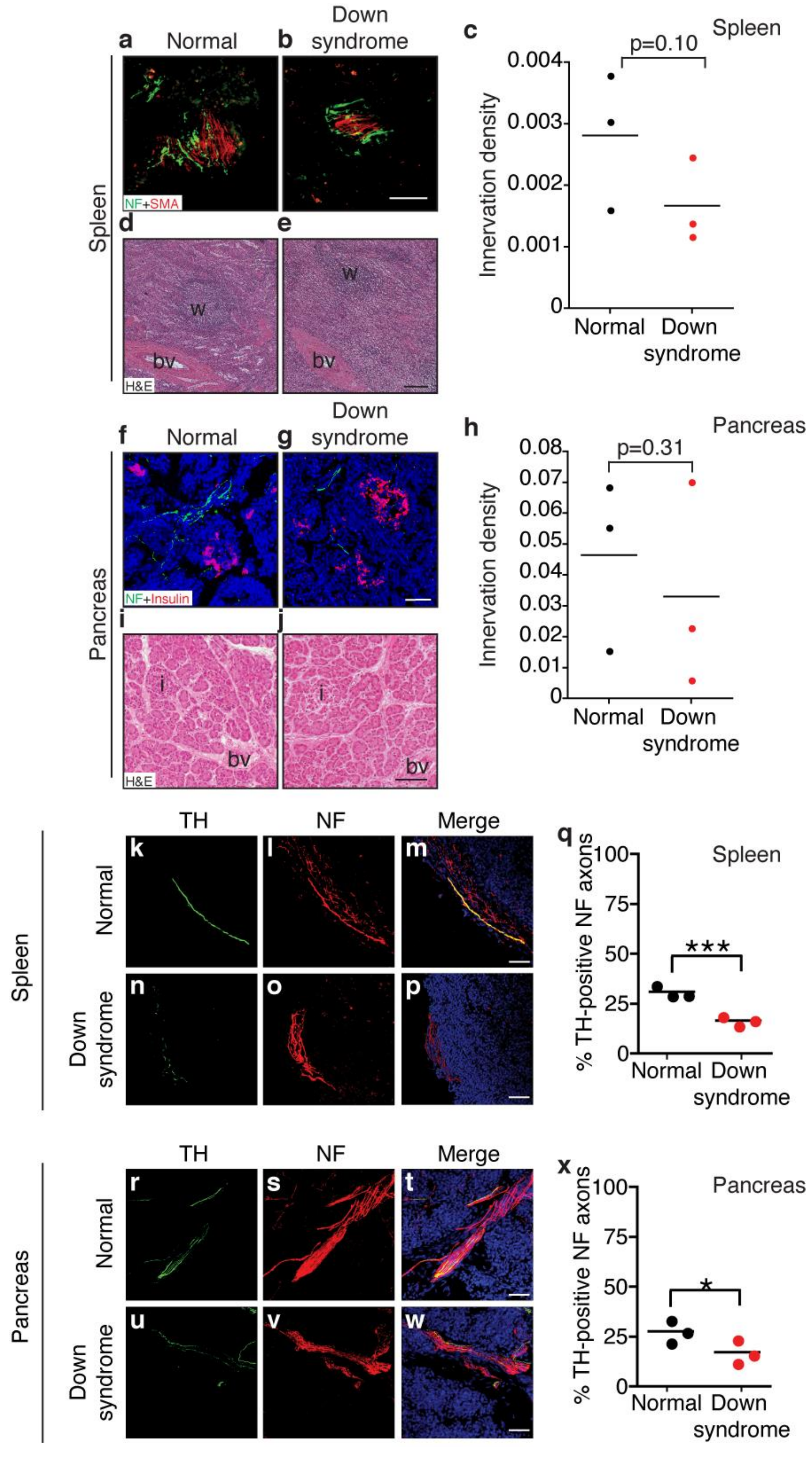
a
Normal
b
Down syndrome
c
Spleen
p=0.10
0.004
0.003
0.002
0.001
0
Innervation density
Normal
Down syndrome
NF+SMA
Spleen
d
e
w
bv
H&E
f
Normal
g
Down syndrome
h
Pancreas
p=0.31
0.08
0.07
0.06
0.05
0.04
0.03
0.02
0.01
0
Innervation density
Normal
Down syndrome
NF+Insulin
Pancreas
i
j
i
bv
H&E
TH
NF
Merge
k
l
m
Normal
Spleen
n
o
p
Down syndrome
q
Spleen
100
75
50
25
0
% TH-positive NF axons
***
Normal
Down syndrome
TH
NF
Merge
r
s
t
Normal
Pancreas
u
v
w
Down syndrome
x
Pancreas
100
75
50
25
0
% TH-positive NF axons
*
Normal
Down syndrome

Supplementary Figure 2. Analyses of innervation in human Down syndrome tissues

(**a-j**) Gross morphology of human Down syndrome tissues is comparable to matched controls, but overall innervation is slightly reduced. (**a-c, f-h**) Neurofilament immunostaining (in green) in the spleen (**a-c**) and pancreas (**f-h**) shows a slight decrease in overall innervation of Down syndrome tissues. Spleen tissue sections were immunostained with smooth muscle actin (SMA, red) for blood vessels, and pancreatic tissues were immunostained with insulin (in red), and counter-stained for DAPI (blue). Scale bar: 50 µm (**c, h**) Quantification of neurofilament immunoreactivity in the spleen and pancreatic tissues. Results are expressed as fluorescence units per 100 μm^2. n=3 Down syndrome and 3 control donors. p values are 0.10 and 0.31 for spleen and pancreas, respectively, using one tailed *t*-test. (**d-e, i-j**) The gross morphology of Down syndrome spleen (**e**) and pancreas (**j**), as visualized by H&E staining, appear similar to control tissues. bv=blood vessels, w=white pulp, i=islet. Scale bar: 50 µm. (**k-x**) Co-immunostaining for Tyrosine Hydroxylase (TH) and Neurofilament (NF) in human peripheral tissues. A significant decrease in the proportion of TH/NF double-positive axons is observed in the spleen (**k-q**) and pancreas (**r-x**) from Down syndrome individuals compared to control individuals. TH (green), NF (red) and DAPI in blue. n=3 Down syndrome and 3 control donors. ***p=0.0013 for spleen and *p=0.0458 for pancreas, *t*-test. Scale bar: 50 µm.

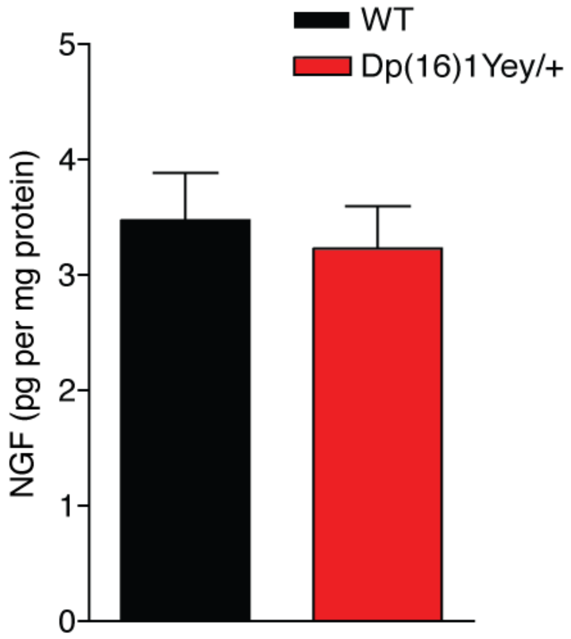

Supplementary Figure 3. NGF production is unaffected in *Dp(16)1Yey/+* peripheral tissues

NGF protein levels are similar in heart lysates from P1.5 *Dp(16)1Yey/+* mice and wild-type litter-mates, as determined using an ELISA assay. Results are the mean ± SEM from n=7 mice per genotype. p=0.667, *t*-test, unpaired, two-tailed.

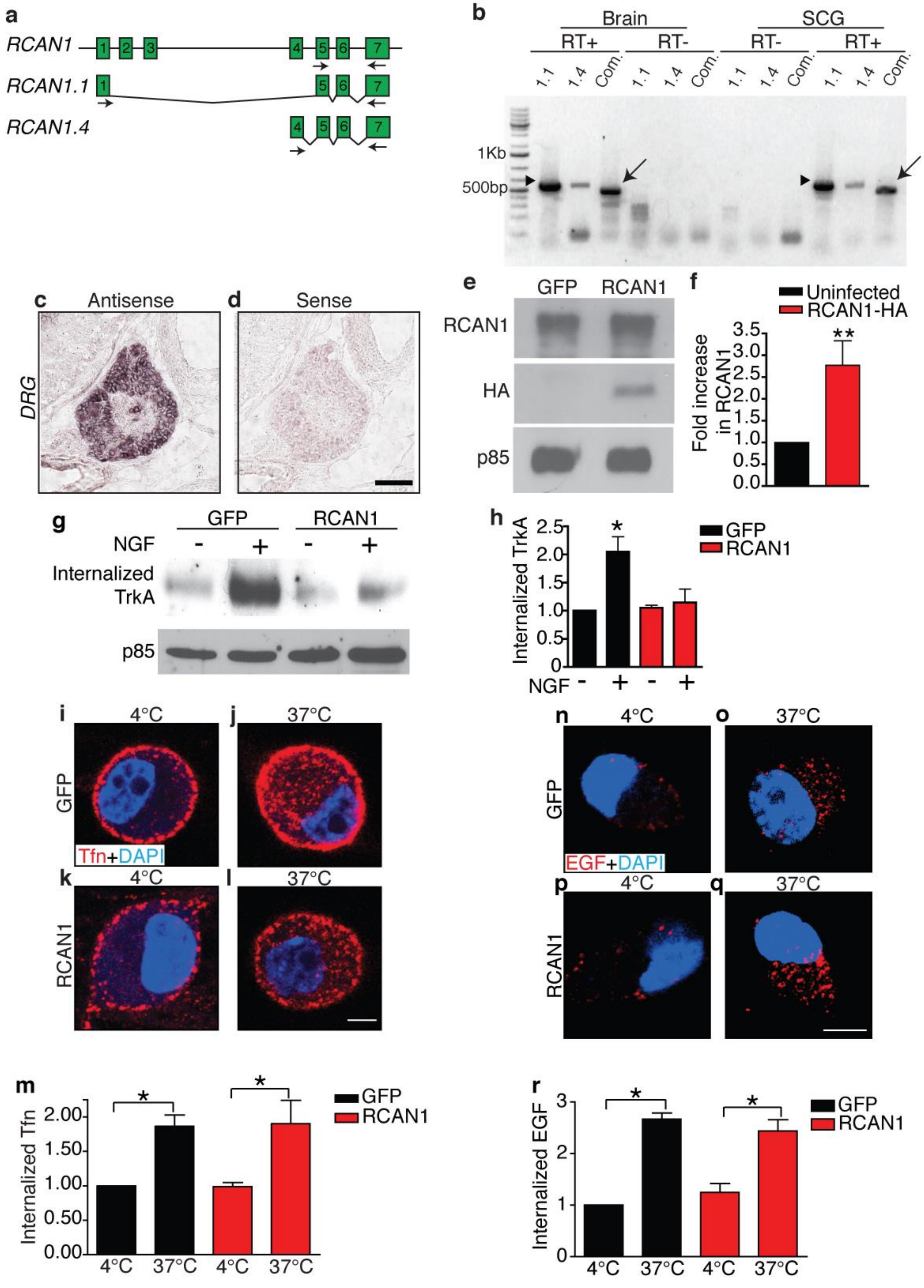
a
RCAN1
RCAN1.1
RCAN1.4
b
Brain
SCG
RT+
RT-
1.1
1.4
Com.
1Kb
500bp
c
Antisense
d
Sense
DRG
e
GFP
RCAN1
HA
p85
f
Uninfected
RCAN1-HA
Fold increase in RCAN1
g
NGF
Internalized TrkA
h
Internalized TrkA
i
4°C
j
37°C
Tfn+DAPI
k
l
RCAN1
n
o
EGF+DAPI
p
q
m
Internalized Tfn
r
Internalized EGF

Supplementary Figure 4. Analyses of RCAN1 over-expression in peripheral sympathetic and sensory neurons

(a) Schematic representation of *RCAN1.1* and *RCAN1.4* isoforms generated by dual promoter usage. Mouse and human *RCAN1* share the same genomic coding exon organization. Alternative selection of exons 1 and 4 as the first exon results in two different transcripts, with both sharing the last three coding exons. Arrows indicate the position of primers used for RT-PCR analyses. **(b)** Semi-quantitative RT-PCR analysis shows the relative expression of *RCAN1.1* and *RCAN1.4* isoforms in brain and SCG tissues. Arrows and arrow-heads indicate the *RCAN1* bands, as confirmed by sequencing. **(c,d)** *In situ* hybridization shows RCAN1 expression in the developing mouse Dorsal Root Ganglia (DRG) at P0.5. Scale bar: 100 μm. **(e,f)** Adenovirus-mediated over-expression of HA-tagged human RCAN1.4 results in a 2.8-fold increase in RCAN1 protein expression. Mass cultures of sympathetic neurons were infected with either RCAN1.4 or control GFP adenovirus. 48 hr post-infection, neuronal lysates were prepared and immunoblotted for RCAN1. The blot was stripped and reprobed for HA and p85. Densitometric quantification of RCAN1 protein levels normalized to p85. $^{**}p<0.01$; n=6 independent experiments. (**g**) RCAN1 over-expression attenuates NGF-dependent internalization of TrkA receptors in cultured DRG neurons as assessed by a cell surface biotinylation assay. (**h**) Densitometric quantification of internalized TrkA. n=3 independent experiments, $^{*}p<0.05$ different from all other conditions. **(i-m)** Uptake of Alexa-555-labeled Transferrin (Tfn) is unaffected by RCAN1 over-expression in sympathetic neurons. Images were acquired by confocal microscopy and 3-dimensional reconstructions from z-stacks using Image J. Internalized receptors were calculated as Alexa-555-fluorescent pixels per μm^2 of cell body. The values from conditions at 4°C in GFP-expressing neurons were set to 1, and all other conditions are represented relative to the 4°C GFP

values. 40-50 neurons were analyzed per condition per experiment. *p<0.05 relative to 4°C conditions, n=3. Scale bar: 5 μm. (**n-r**) Similarily, internalization of Alex-555-EGF remains unchanged in sympathetic neurons overexpressing RCAN1. 3-dimensional reconstructions from confocal z-stacks were analyzed in Image J. Internalization was analyzed as Alexa-555-fluorescent pixels per μm^2 of cell body. All values are normalized to 4°C in GFP-expressing neurons which was set to 1. 20-30 neurons were analyzed per condition per experiment. *p<0.05 relative to 4°C conditions, n=3. Scale bar: 5 μm. Unpaired two-tailed Student's *t* test for **(f)** and two way ANOVA followed by Bonferroni post-hoc test for **(h, m,r)**.

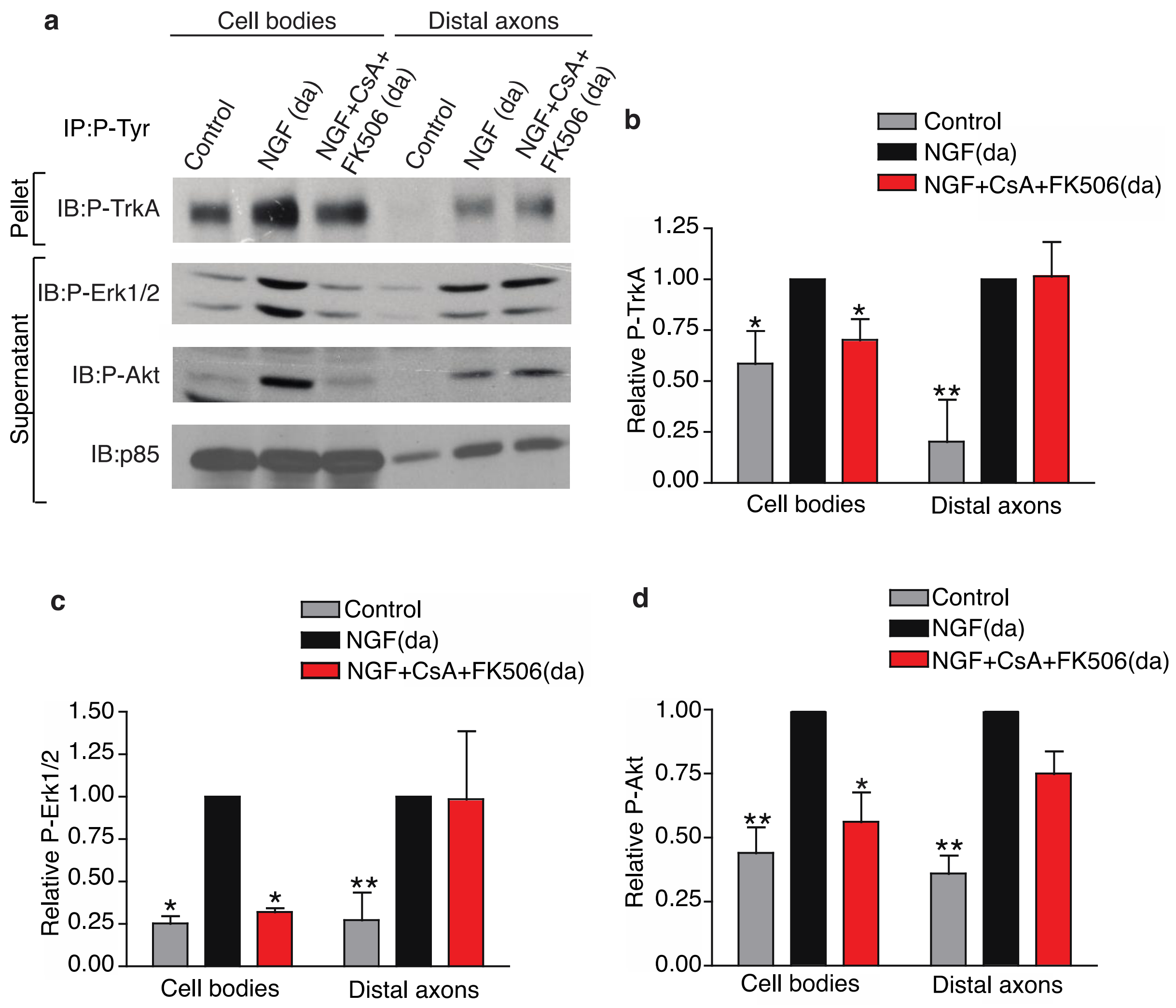

Supplementary Figure 5. Calcineurin activity in distal axons is necessary to propagate a retrograde NGF signal

(a) NGF-dependent retrograde accumulation of P-TrkA, P-Erk1/2 and P-Akt is attenuated by local inhibition of calcineurin activity in distal axons. Distal axons (da) of sympathetic neurons were stimulated with NGF (100 ng per ml) in the presence of the calcineurin inhibitors (CsA and FK506) for 8 hr. Lysates prepared from cell body and distal axon compartments were subjected to P-Tyr immunoprecipitation followed by immunoblotting for TrkA to detect P-TrkA. Supernatants were immunoblotted for P-Erk1/2, P-Akt and p85. **(b-d)** Densitometric

quantifications of levels of P-TrkA **(b)**, P-Erk1/2 **(c)**, and P-Akt **(d),** normalized to p85 levels. Results are means ± SEM from 5 independent experiments and expressed relative to the NGF-stimulated condition. *$p<0.05$, **$p<0.01$ as determined by one-way ANOVA followed by Tukey's post-hoc test.

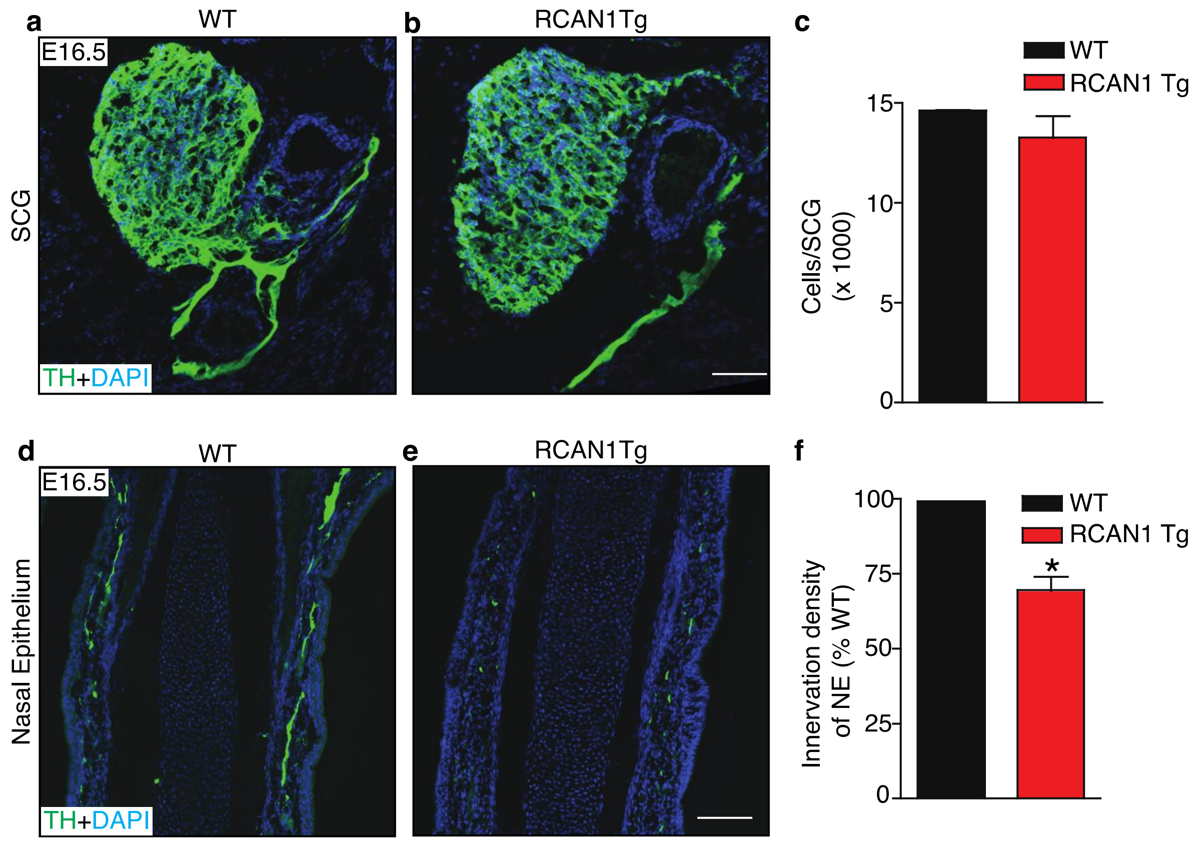

Supplementary Figure 6. Normal SCG neuronal numbers but reduced sympathetic innervation in embryonic *RCAN1* trisomic mice

(a-c) At E16.5, *RCAN1* trisomic mice show comparable SCG size, shape and neuronal numbers as wild-type litter-mates, indicating that early embryonic stages of sympathetic nervous system development are unaffected by excess RCAN1. SCGs were visualized by TH immunohistochemistry and cell counts were performed on Nissl stained tissue sections. Results are means ± SEM from n=3 mice per genotype. **(d-f)** Fewer TH-positive sympathetic fibers were observed innervating the nasal epithlium in E16.5 *RCAN1* trisomic mice compared to wild-type embryos. Quantification of innervation density was estimated from n=3 mice per genotype. Values are the mean ± SEM. The results are represented as a percentage of the mean for wild-type mice. *p<0.05 using unpaired two-tailed *t* test. Scale bar: 50 µm.

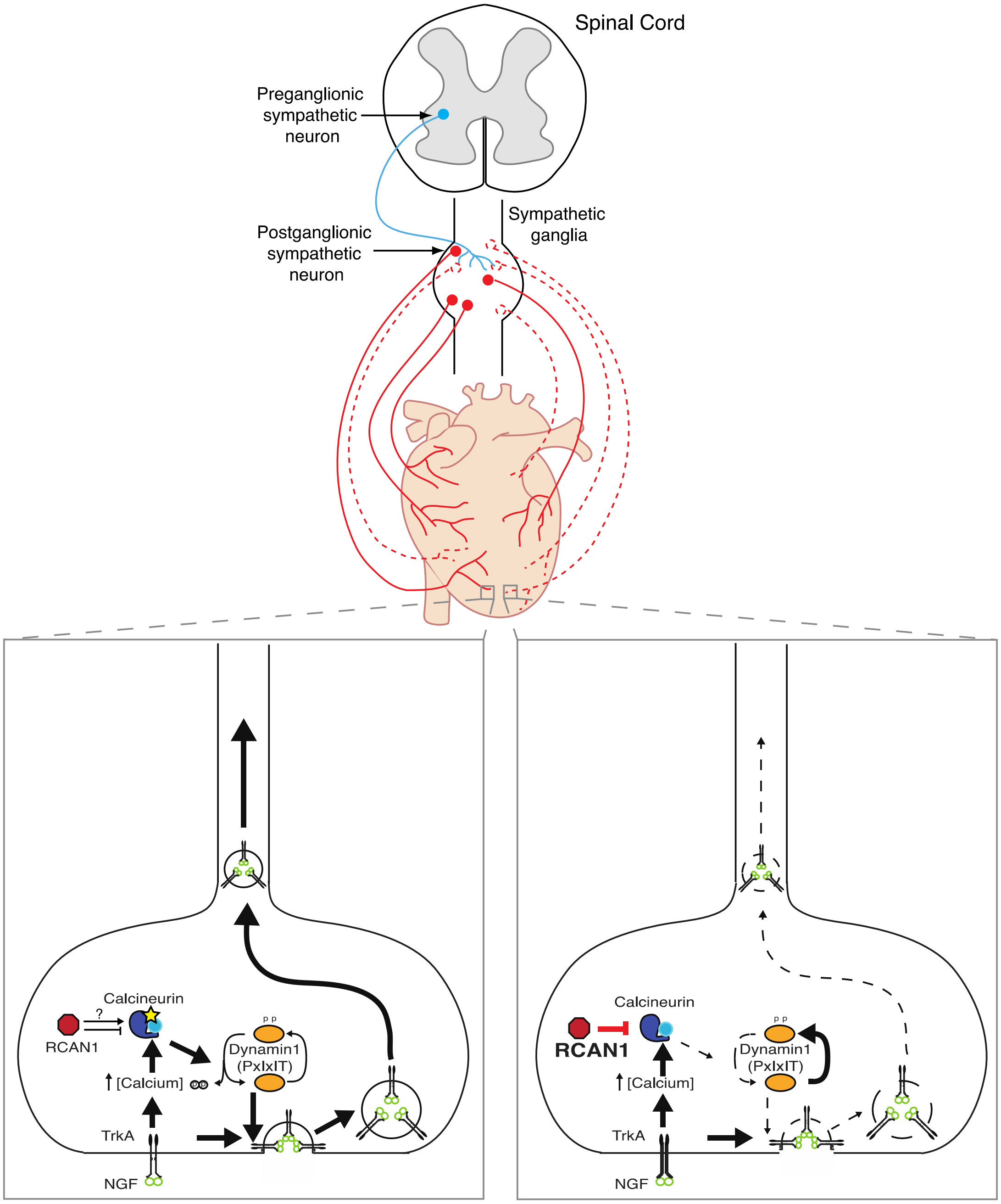

Normal

- TrkA endocytosis
- Retrograde signaling
- Target innervation
- Neuronal survival

Down syndrome

- Inhibition of endocytosis
- Decreased retrograde signaling
- Decreased innervation
- Apoptosis

Supplementary Figure 7. ***RCAN1*** **trisomy links impaired neurotrophin receptor trafficking to neurodevelopmental deficits in Down syndrome**

Post-ganglionic sympathetic neurons project to diverse peripheral organs and tissues to regulate tissue homeostasis, in a manner dependent on the target-derived neurotrophic factor, NGF, during development. NGF promotes endocytosis of TrkA receptors in nerve terminals by activating calcineurin-mediated dephosphorylation of neuron-specific splicing isforms of dynamin1 that harbor a calcineurin interaction PxIxIT motif. NGF:TrkA-containing signaling endosomes are then retrogradely transported back to neuronal cell bodies to initiate transcriptional programs necessary for neuronal survival and growth. In Down Syndrome, over-expression of RCAN1, an endogenous calcineurin inhibitor, interferes with NGF-mediated phosphoregulation of dynamin1, internalization of TrkA receptors and retrograde trophic signaling. The loss of target-derived neurotrophic support contributes to diminished sympathetic innervation of target tissues and enhanced apoptosis of NGF-responsive sympathetic neurons in Down syndrome.

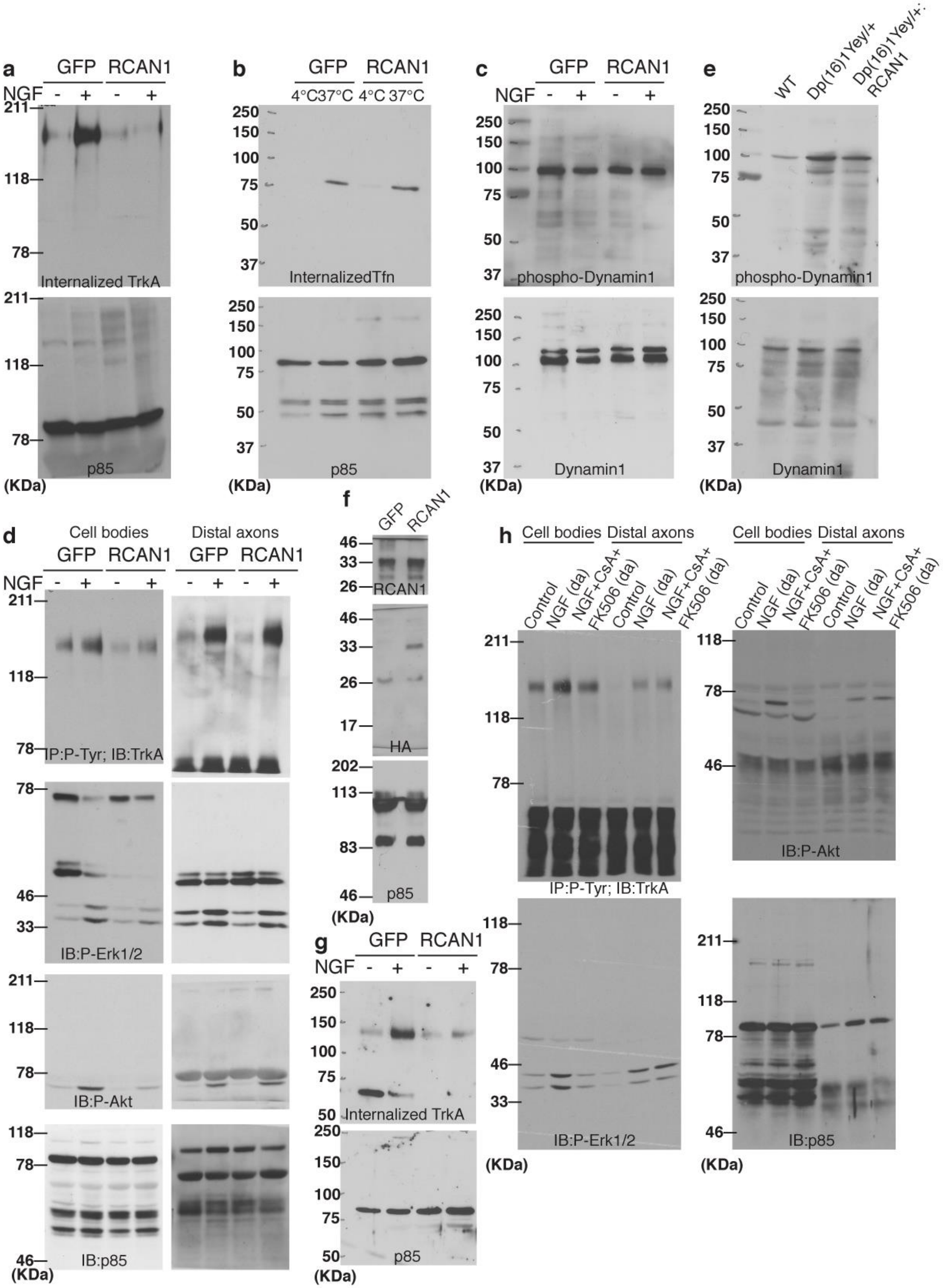
a
GFP RCAN1
NGF - + - +
Internalized TrkA
p85
(KDa)
b
GFP RCAN1
4°C 37°C 4°C 37°C
InternalizedTfn
p85
(KDa)
c
GFP RCAN1
NGF - + - +
phospho-Dynamin1
Dynamin1
(KDa)
e
WT
Dp(16)1Yey/+
Dp(16)1Yey/+: RCAN1
phospho-Dynamin1
Dynamin1
(KDa)
d
Cell bodies
Distal axons
GFP RCAN1 GFP RCAN1
NGF - + - + - + - +
IP:P-Tyr; IB:TrkA
IB:P-Erk1/2
IB:P-Akt
IB:p85
(KDa)
f
GFP RCAN1
RCAN1
HA
p85
(KDa)
g
GFP RCAN1
NGF - + - +
Internalized TrkA
p85
(KDa)
h
Cell bodies Distal axons
Control
NGF (da)
NGF+CsA+ FK506 (da)
IP:P-Tyr; IB:TrkA
IB:P-Erk1/2
IB:P-Akt
IB:p85
(KDa)

Supplementary Figure 8. Full-length immunoblots

(a) Full-length blots for Figure 3f. **(b)** Full-length blots for Figure 3h. **(c)** Full-length blots for Figure 3j. **(d)** Full-length blots for Figure 4b. **(e)** Full-length blots for Figure 7c. **(f)** Full-length blots for Supplementary Figure 4e. **(g)** Full-length blots for Supplementary Figure 4g. **(h)** Full-length blots for Supplementary Figure 5a.

Donor Tissue	NBB ID	Sex	Age (days)	Cause of Death
Normal 1 (spleen, pancreas)	82	M	137	Pneumonia/apnea
Normal 2 (spleen, pancreas)	390	F	125	Asphyxia
Normal 3 (spleen)	461	F	415	Asphyxia
Normal 4 (pancreas)	440	M	667	Unclear
Down syndrome 1 (spleen, pancreas)	832	M	339	Cardiac arrhythmia
Down syndrome 2 (spleen, pancreas)	1282	F	186	Heart defect
Down syndrome 3 (spleen)	718	F	427	Pneumonia
Down syndrome 4 (pancreas)	714	M	684	Cardiac arrhythmia

Supplementary Table 1: Information of the tissue samples from Down syndrome and normal individuals.
